# Elucidating the Role of O_2_ Uncoupling in the Oxidative Biodegradation of Organic Contaminants by Rieske Non-heme Iron Dioxygenases

**DOI:** 10.1021/acsenvironau.2c00023

**Published:** 2022-07-07

**Authors:** Charlotte E. Bopp, Nora M. Bernet, Hans-Peter E. Kohler, Thomas B. Hofstetter

**Affiliations:** † Eawag, Swiss Federal Institute of Aquatic Science and Technology, 8600 Dübendorf, Switzerland; ‡ Institute of Biogeochemistry and Pollutant Dynamics (IBP), ETH Zürich, 8092 Zürich, Switzerland

**Keywords:** non-heme ferrous iron oxygenases, Rieske oxygenases, biocatalysis, O_2_ uncoupling, O_2_ activation, kinetic isotope effect, biodegradation

## Abstract

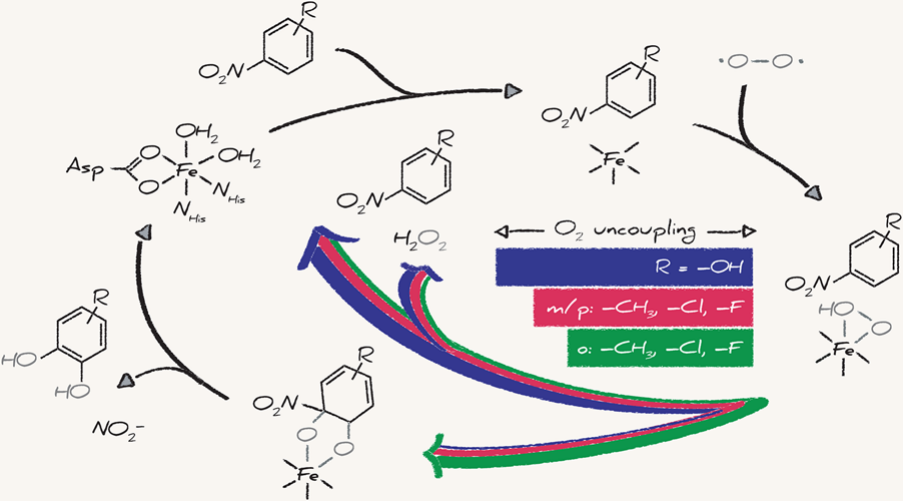

Oxygenations of aromatic soil and water contaminants with molecular O_2_ catalyzed by Rieske dioxygenases are frequent initial steps of biodegradation in natural and engineered environments. Many of these non-heme ferrous iron enzymes are known to be involved in contaminant metabolism, but the understanding of enzyme–substrate interactions that lead to successful biodegradation is still elusive. Here, we studied the mechanisms of O_2_ activation and substrate hydroxylation of two nitroarene dioxygenases to evaluate enzyme- and substrate-specific factors that determine the efficiency of oxygenated product formation. Experiments in enzyme assays of 2-nitrotoluene dioxygenase (2NTDO) and nitrobenzene dioxygenase (NBDO) with methyl-, fluoro-, chloro-, and hydroxy-substituted nitroaromatic substrates reveal that typically 20–100% of the enzyme’s activity involves unproductive paths of O_2_ activation with generation of reactive oxygen species through so-called O_2_ uncoupling. The ^18^O and ^13^C kinetic isotope effects of O_2_ activation and nitroaromatic substrate hydroxylation, respectively, suggest that O_2_ uncoupling occurs after generation of Fe^III^-(hydro)peroxo species in the catalytic cycle. While 2NTDO hydroxylates *ortho*-substituted nitroaromatic substrates more efficiently, NBDO favors *meta*-substituted, presumably due to distinct active site residues of the two enzymes. Our data implies, however, that the O_2_ uncoupling and hydroxylation activity cannot be assessed from simple structure–reactivity relationships. By quantifying O_2_ uncoupling by Rieske dioxygenases, our work provides a mechanistic link between contaminant biodegradation, the generation of reactive oxygen species, and possible adaptation strategies of microorganisms to the exposure of new contaminants.

## Introduction

Oxygenations of aromatic and aliphatic hydrocarbons with molecular O_2_ are a frequent initial step of the biodegradation of anthropogenic organic contaminants.^1,2^ The oxygenated products are often more polar and more bioavailable than the substrate and can be transformed further in standard metabolic pathways that support microbial growth and energy metabolism.^3,4^ Enzymatic oxygenations of recalcitrant aromatic contaminants from a wide range of applications and origins, including pharmaceuticals, industrial chemicals, and explosives,^5−14^ are all catalyzed by Rieske dioxygenases (RDOs), a subgroup of non-heme ferrous iron oxygenases involved in many catabolic and biosynthetic processes.^15−29^ Even though many contaminant-degrading RDOs are well-known, the factors that determine which enzyme-contaminant combinations lead to successful substrate oxygenation and at which rate contaminant transformation occurs are largely unknown. A generalized assessment of this important reaction path for contaminant biodegradation is therefore hardly possible.

**Scheme 1:**
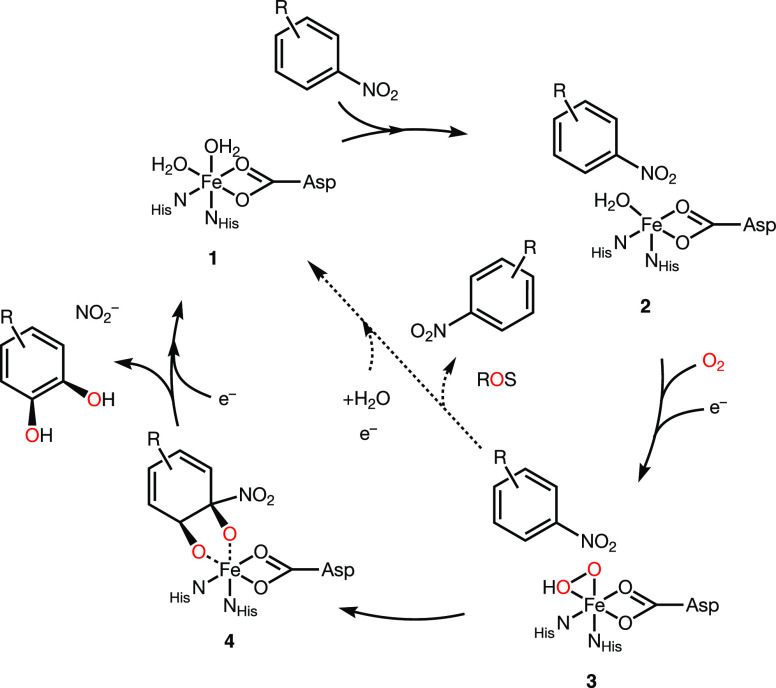
Catalytic Cycle of Nitrobenzene Dioxygenase Shown As Model for Non-Heme Ferrous Iron Rieske Dioxygenases In its resting state (**1**), the non-heme Fe is six-coordinate. The presence of the substrate triggers Fe coordination changes (**2**) required for O_2_ activation and electron transfer from the Rieske cluster (**3**), shown here as an arbitrary Fe-hydroperoxo species. Activated O_2_ is utilized productively in the formation of the dihydroxylated product (**4**) or unproductively in the release of reactive oxygen species (ROS).

In fact, the role of substrates in the catalytic cycles and kinetic mechanisms of RDOs is still elusive except those used in the characterization of the two prototypical enzymes naphthalene and benzoate dioxyxgenase.^30−32^ In contrast to other non-heme ferrous iron oxygenases, RDOs retrieve only two of the four electrons required for the reduction of O_2_ from the substrate.^18,22,23^ Two additional reduction equivalents originate from NADH oxidation and are supplied through electron transfer proteins via the Rieske cluster.^11,33,34^ Hydroxylation of the substrate and, thus, contaminant transformation are preceded by a series of steps responsible for enzymatic O_2_ activation (Scheme 1) for which the role of the substrate is hardly known.^35^ RDOs do not bind the substrate to the non-heme Fe center but require their presence in the substrate binding pocket to induce coordination changes at the non-heme Fe (**1** → **2**, Scheme 1), followed by O_2_ binding and electron transfer from the Rieske cluster (**2** → **3**).^30,35^ Hydroxylations of aromatic moieties are then carried out by (high-valent) Fe-oxygen species (**3** → **4**) which have been assigned to superoxo-, peroxo-, and oxo species.^32,36−38^ While substrates exert some allosteric control on O_2_ activation to Fe-oxygen species in RDOs, the substrate is not directly involved in these rate-limiting steps of the catalytic cycle.^31,32,38−40^ An assessment of the reactivity of RDOs toward different substrates on the basis of contaminant transformation rates therefore appears somewhat arbitrary.

An often overlooked aspect of catalytic cycles of contaminant-degrading RDOs as well as other O_2_ activating enzymes is the unproductive activation of O_2_ that generates and releases reactive oxygen species (ROS) from the active site without oxidation of the substrate. Despite being a well-known phenomenon in the activity of non-heme ferrous iron oxygenases,^41−47^ this so-called O_2_ uncoupling and its consequences for assessing contaminant biotransformation remain largely unexplored (see compilation in Bopp et al.^11^). Uncoupling of activated O_2_ can have three principal consequences. First, release of ROS from the active site can be associated with hydroxylation of electron-rich amino acid side chains such as tryptophan and tyrosine residues of the oxygenase itself.^41^ Such protein hydroxylations are typically associated with a loss of enzyme activity. Second, a reconfiguration of metabolic fluxes is observed upon ROS release from the oxygenase^48^ as part of defense and repair mechanisms of various cell components such as lipids, enzymes, and nucleic acids.^49−51^ Qualitatively, such an oxidative stress response has been observed repeatedly in ring-hydroxylating bacteria upon exposure to aromatic compounds^52−54^ and involves the consumption of reduction equivalents also used in contaminant oxygenation reactions. Finally, O_2_ uncoupling and concomitant formation of ROS have been associated with interferences in the regulation and expression of genes encoding for RDOs, thereby accelerating the enzymatic adaptation toward new substrates.^55−57^ Despite the various consequences of O_2_ uncoupling on the microbial capability to initiate biodegradation through oxygenation reactions, an understanding of the extent and catalytic mechanism of this process upon exposure of RDOs to different aromatic contaminants is lacking. Given that microbes are exposed to mixtures of organic contaminants in the environment, it would be important to know whether O_2_ uncoupling is an innate consequence of the broad substrate specificity of RDOs or whether it is triggered by properties of the substrates that lead, for example, to a bad fit in the active site and ensuing changes in geometric and electronic structures of Fe-oxygen species.^45^

The objective of this work was to evaluate the relevance of O_2_ uncoupling for the dioxygenation of aromatic substrates by RDOs and to provide a mechanistic basis to account for this process when assessing contaminant biodegradation. Here, we studied two important and well-characterized nitroarene dioxygenases, 2-nitrotoluene dioxygenase (2NTDO) and nitrobenzene dioxygenase (NBDO), as representative RDOs.^40,58−64^ We obtained insights into the substrate- and enzyme-specificity of O_2_ uncoupling in a comprehensive evaluation of the activity of 2NTDO as well as through extension of a previous data set for NBDO.^65^ The specific goals were as follows. (1) We aimed to quantify the extent of O_2_ uncoupling for a wide set of structurally related substrates of nitroarene dioxygenases on the basis of in vitro enzyme assays. 2NTDO and NBDO share 95% sequence identity and cover a similar substrate spectrum,^64^ yet two distinct active site residues have been found to alter the enzymes’ substrate specificity.^66^ (2) We elucidated the catalytic mechanism of nitroarene dioxygenases to characterize the elementary reactions responsible for O_2_ uncoupling by RDOs. To that end, we studied kinetic isotope effects of both substrates, O_2_ and nitroaromatic compounds, to probe for the mechanisms and timing of their reactions in the catalytic cycle. While ^18^O kinetic isotope effects (^18^O-KIEs) were used to infer the type of reactive Fe-oxygen species formed,^67−73^^13^C-KIEs allowed for studying the initial step of aromatic hydroxylation.^40,61,74^ (3) We examined the influence of substrate molecular structure on the oxygenation reaction by comparing the extent of O_2_ uncoupling for a broad set of methylated, hydroxylated, fluorinated, and chlorinated nitroaromatic substrates. Finally, we rationalize wider implications of O_2_ uncoupling scrutinized here for two RDOs for assessing oxidative contaminant biodegradation in the environment.

## Experimental Section

All chemicals and material used are reported in section S1 in the Supporting Information (SI). Enzyme purification procedures were largely adapted from previous works^60,75,76^ as described in section S2. Experimental procedures follow methods described by Pati and co-workers^61,65^ and are summarized in the following.

### Enzyme Assays

#### Controlled Substrate Turnover Experiments

We quantified the turnover of nitroaromatic substrates to organic and inorganic reaction products (substituted catechols, benzylic alcohols, and nitrite) as well as O_2_ disappearance from a single set of enzyme assays where the reaction progress was controlled through the amount of NADH added. The same samples were also used for determination of organic substrate ^13^C/^12^C and ^18^O/^16^O ratios of dissolved O_2_. Due to the amounts of O_2_ required for ^18^O/^16^O ratio measurements in gaseous O_2_,^77,78^ these assays were set up in 12 mL clear-glass crimp-top vials. Each vial contained a magnetic stir bar and was filled completely (i.e., without headspace) and closed with butyl rubber aluminum crimp seals. Experiments were carried out in 50 mM MES buffer (pH 6.8) equilibrated at room temperature (20–25 °C) to obtain initial dissolved O_2_ concentrations of 220–280 μM. Assays consisted of 0.15 μM reductase, 1.8 μM ferredoxin, 0.15 μM oxygenase, 100 μM (NH_4_)_2_Fe(SO_4_)_2_, and 40–170 μM nitroaromatic substrate added from 50 mM methanolic stock solutions. Purified oxygenase was thawed directly before the experiment, whereas ferredoxin and reductase were kept in the refrigerator for up to 1 week. Reactions were initiated by the addition of 10–50 μL of 50 mM NADH stock (in 10 mM NaOH) with a gastight glass syringe through the septum of the closed vials. NADH concentrations of stock solutions were determined spectrophotometrically (ϵ_340 nm_= 6300 L mol^–1^ cm^–1^).^79^ For each enzyme–substrate combination, four to six replicate experiments, each with a different initial NADH concentration (20–330 μM), were performed in separate reactors. Dissolved O_2_ concentrations were monitored continuously with a needle-type oxygen microsensor (PreSens, Precision Sensing GmbH) immersed into the assay under constant stirring of the sample at 300 rpm. Reactions were run until complete oxidation of NADH which became evident from spectrophotometric measurements of NADH as well as from the observation of O_2_ concentration leveling off at constant concentrations after 5–40 min. Initial nitroaromatic substrate concentrations were determined in sample vials with substrate in MES buffer in the absence of any enzyme. Background consumption of O_2_ in enzyme assays was monitored and assessed systematically as described in section S3.1.

#### Quantification of H_2_O_2_

We quantified H_2_O_2_ formation for a selected number of enzyme–substrate combinations in separate enzyme assays where horse radish peroxidase (HRP) was used to catalyze the reduction of H_2_O_2_ with concomitant oxidation of 4-methoxyaniline or 10-acetyl-3,7-dihydroxyphenoxazine (Ampliflu).^80,81^ Losses of 4-methoxyaniline or Ampliflu provided a measure for the amount of H_2_O_2_ formed.

In assays with NBDO and 2- and 4-nitrotoluene, H_2_O_2_ was quantified from aliquots of controlled turnover assay described above. After complete NADH oxidation, 900 μL aliquots were withdrawn and mixed with 100 μL of an HRP assay in MES buffer resulting in final concentrations of 10 mg L^–1^ HRP and 500 μM 4-methoxyaniline. 4-Methoxyaniline consumption was quantified on HPLC as described in section S3.2.1 and an external calibration row of 4-methoxyaniline consumption by HRP with a range of H_2_O_2_ concentrations of 50–250 μM.

For experiments with 2NTDO, we prepared separate assays for the quantification of H_2_O_2_ formation with nitrobenzene, 2-nitrotoluene, as well as the three chloronitrobenzene isomers. The assays were prepared in 2 mL crimp vials filled completely with MES buffer containing 0.15 μM reductase, 1.8 μM ferredoxin, 0.15 μM oxygenase, 100 μM (NH_4_)_2_Fe(SO_4_)_2_, and 300 μM of nitroaromatic substrate. Substrate oxygenations were initiated by addition of 100–200 μM of NADH through the septum and run with continuous stirring and O_2_ monitoring until O_2_ concentrations remained constant. Subsequently, 900 μL aliquots were mixed with 100 μL of the above-mentioned HRP assay in MES buffer (10 mg L^–1^ HRP and 400 μM Ampliflu). Ampliflu was quantified spectrophotometrically at 560 nm on a plate reader (Synergy Mx, Biotek Instruments Inc., Vermont, VT, USA) and an external calibration row of Ampliflu with a range of H_2_O_2_ concentrations from 20 to 250 μM.^80^

#### Kinetics of Enzymatic O_2_ Consumption

The kinetics of O_2_ consumption were determined in 2 mL crimp vials equipped with a magnetic stir bar (300 rpm) at approximately 22 °C and filled completely with enzyme assay solution following procedures established by Pati et al.^65^ All assays contained slightly modified concentrations to prevent anything but O_2_ availability limiting turnover (0.3 μM reductase, 3.6 μM ferredoxin, 0.15 μM oxygenase, 500 μM (NH_4_)_2_Fe(SO_4_)_2_), and experiments were run in excess of nitroaromatic substrate (500 μM). Reactions were initiated through the addition of NADH from a 100 mM stock solution through the septum to obtain a final concentration of 1000 μM. All experiments were run until complete consumption of dissolved O_2_ (250 μM).

#### Substrate Oxygenation Kinetics from NO_2_^–^ Formation

The initial rates of NO_2_^–^ formation from nitrobenzene, 2-nitrotoluene, and 3-chloronitrobenzene were determined in triplicate at six different initial substrate concentrations ranging from 10 to 300 μM. Experiments were performed at room temperature (approximately 20 °C) in 1.5 mL plastic tubes containing 0.5 mL of MES buffer (50 mM, pH 6.8) with 0.3 μM reductase, 3.6 μM ferredoxin, 0.15 μM oxygenase, and 500 μM (NH_4_)_2_Fe(SO_4_)_2_. The reaction was initiated by the addition of 500 μM NADH, and 100 μL samples were withdrawn after 20, 30, 40, and 50 s. The reaction was quenched with 200 μL of sulfanilamide (10 g L^–1^ in 1.5 M HCl) followed by the addition of 200 μL of *N*-(1-naphthyl)ethylenediamine dihydrochloride (1 g L^–1^ in 1.5 M HCl). NO_2_^–^ was quantified using a photometric method at 540 nm^82^ with an external calibration exhibiting standard deviations of <3 μM.

### Chemical and Isotopic Analyses

#### Quantification of Organic Substrate and Product Concentrations

Organic substrates, nitrobenzylalcohols, and catecholic products were quantified by HPLC as described in detail in section S3.2.1.

#### Stable Isotope Analyses

After completion of controlled substrate turnover experiments, the 12 mL vials were prepared for analysis of ^18^O/^16^O ratios in O_2_ according to procedures described previously.^61,77,78^ Briefly, 3 mL of the assay solution was removed with a gastight syringe by simultaneously filling the vial with N_2_ gas (5.0) at a constant pressure of 2 bar. The reactors were placed upside down on an orbital shaker at 200 rpm for 30 min to accelerate partitioning of O_2_ into the headspace. Then 1000 μL of gaseous headspace was injected into a gas chromatograph coupled via a Conflo IV interface to an isotope ratio mass spectrometer (GC/IRMS, Thermo Fisher Scientific). Duplicate injections of three samples were bracketed by three injections of ambient air that served as a reference standard for δ^18^O values reported vs Vienna Standard Mean Ocean Water (VSMOW). The δ^18^O values of the reference gas was calibrated with O_2_ signals from on-column injections of air assuming a constant δ^18^O_air_ of 23.88‰.^83^ Instrument parameters were reproduced according to Bopp et al.^78^ with either two connected PLOT columns (Restek from BGB Analytik; 30 m × 0.32 mm ID, 30 μ m film thickness) or a single column employing a linear correction factor to exclude Ar interference in the measurement of ^18^O/^16^O isotope ratios. Each sequence included three blank samples of O_2_-free water that was obtained from 20 min of purging under a constant stream of N_2_ and treated similarly to the samples to account for diffusive O_2_ contamination.^84^

Carbon isotope ratios (^13^C/^12^C) of organic substrates were determined from the 3 mL aqueous samples withdrawn from the 12 mL vials for generation of the N_2_ headspace. Nitroaromatic compounds were extracted from aqueous samples by solid phase microextraction (SPME) and analyzed for ^13^C/^12^C ratios on a GC/IRMS equipped with a GC combustion III interface. Instrumental procedures were described in detail in refs (40) and (61). Samples were diluted to substrate concentrations that resulted in constant peak amplitudes between 0.5 and 8 V. Triplicate measurements of three samples were bracketed by three injections of calibrated in-house reference materials spanning δ^13^C values between −55‰ and +7.7‰ to ensure accuracy of the measurements. δ^13^C values are reported relative to Vienna PeeDee Belemnite (δ^13^C_VPDB_).

### Data Evaluation

#### Reaction Stoichiometries

**Figure 1:**
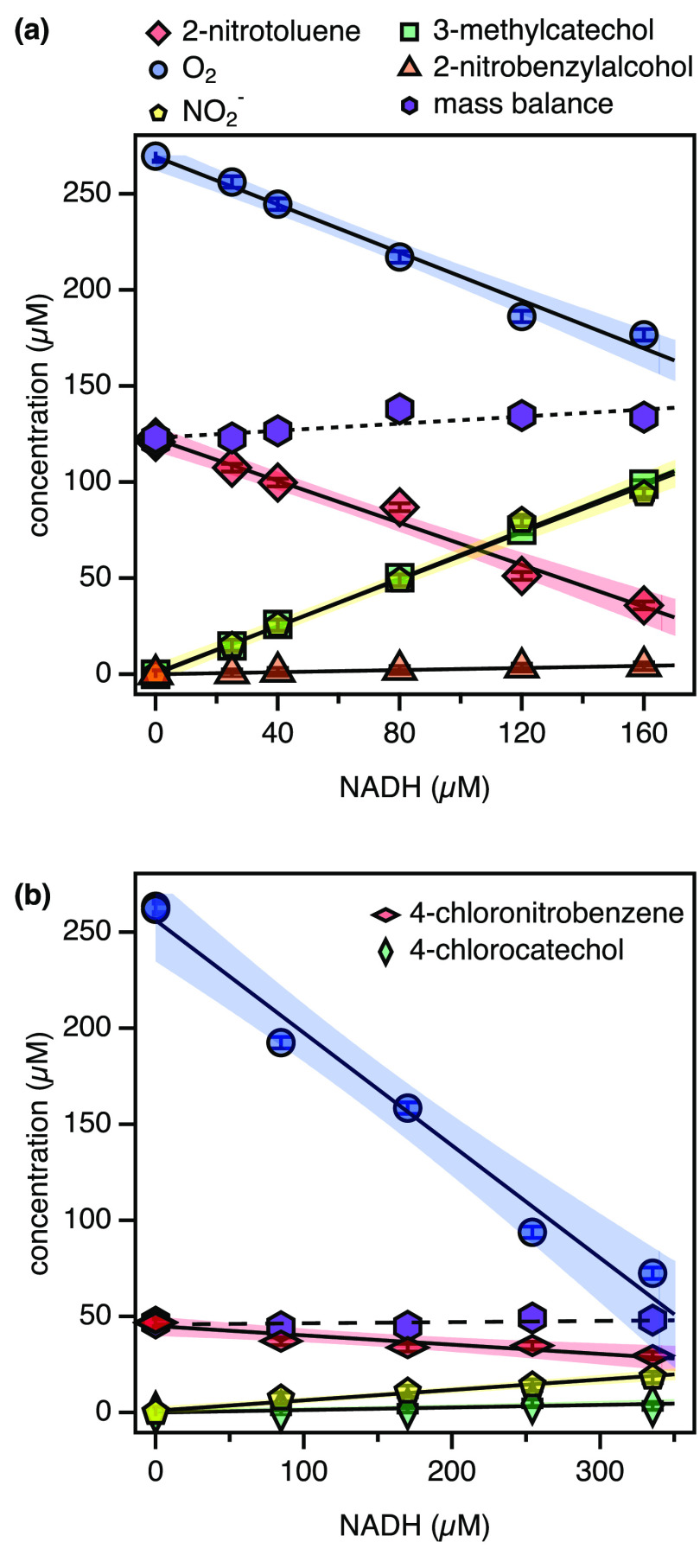
Concentrations of substrate, dissolved O_2_, organic products, and NO_2_^–^ in 2NTDO assays after complete consumption of different amounts of NADH. The black lines and shaded areas represent linear fits with 95% confidence intervals with slopes shown in Table S4. With 2-nitrotoluene as the substrate (a), the mass balance represents the concentrations of 2-nitrotoluene, NO_2_^–^, and 2-nitrobenzylalcohol. For 4-chloronitrobenzene (CNB) as the substrate (b), the mass balance represents the concentrations of 4-chloronitrobenzene and NO_2_^–^.

Reaction stoichiometries of substrate consumption and product formation were normalized to the amount of external reduction equivalents (NADH) of five to eight replicate experiments. Stoichiometric coefficients of species *j*, |υ_*j*_|, were calculated through linear regressions of eq 1 for the different concentrations of nitroaromatic substrate, dissolved O_2_, hydroxylated aromatic product, and NO_2_^–^ obtained from experiments with different amounts of added NADH.
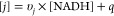
1where [*j*] is the measured molar concentration of substrate, dissolved O_2_, hydroxylated organic product, or nitrite at the end of an experiment, [NADH] is the nominal concentration of NADH, and *q* is the *y*-intercept (Figure 1). Uncertainties of |υ_*j*_| reflect errors arising from linear regression analysis and are reported as 95% confidence intervals.

The extent of O_2_ uncoupling, *f*_O_2_-uc_, was calculated through linear regressions of eq 2: 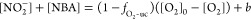
2where [NO_2_^–^] is the concentration of nitrite formed, [O_2_]_0_ is the initial O_2_ concentration, [O_2_] is the residual O_2_ concentration, and [NBA] is the concentration of nitrobenzylalcohol formed by monooxygenation. Figure S3 illustrates regressions for the derivation of O_2_ uncoupling for substrates with efficient and inefficient oxygenation of 2-nitrotoluene and 4-chloronitrobenzene, respectively. Procedures for evaluation of and accounting for background consumption of O_2_ in enzyme assays are documented in section S3.1.

#### Isotope Effects

Apparent kinetic isotope effects pertinent to the hydroxylation of aromatic carbon, ^13^C-KIE, were derived from nonlinear correlations of fractional amount of residual substrate vs the observable changes in ^13^C/^12^C ratios and are expressed in terms of C isotope signatures, δ^13^C, and C isotope enrichment factors, ϵ_C_, according to eqs 3 and 4.
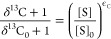
3
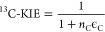
4where δ^13^C and δ^13^C_0_ are the C isotope signatures of the substrate in an experiment vs its original value, respectively. [S] and [S]_0_ are the residual and initial substrate concentrations, respectively. *n*_C_ is the number of carbon atoms in the substrate, which accounts for the isotopic dilution of the isotope effect based on the assumption of an asynchronous hydroxylation mechanism.^31,61^ Nonlinear regression fit weighted with the standard deviation of triplicate measurements were carried out in Igor Pro (WaveMetric Inc.). Note that in cases of substantial O_2_ uncoupling, when substrate turnover was below 30% and changes in δ^13^C of the substrates remained within the total uncertainty of ^13^C/^12^C ratio measurements of 0.5‰, ^13^C-KIE were set to unity (section S3.4).

Kinetic isotope effects associated with O_2_ activation by nitroarene dioxygenases, ^18^O-KIE, were derived as average for both O_2_ atoms in O_2_ according to eq 5 following the identical procedures as outlined above.
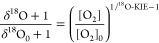
5where [O_2_] and [O_2_]_0_ are the residual and initial dissolved O_2_ concentrations, respectively.

## Results and Discussion

### Efficiency of Substrate Oxygenation by 2-Nitrotoluene Dioxygenase

**Scheme 2:**
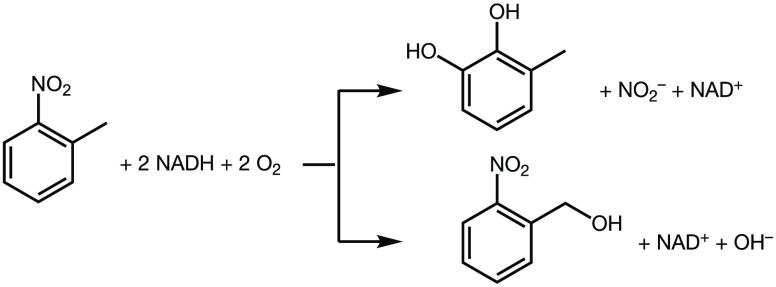
Reactions Catalyzed by 2-Nitrotoluene Dioxygenase

2-Nitrotoluene dioxygenase carries out hydroxylations of nitroaromatic substrates with the concomitant oxidation of NADH for O_2_ activation.^64^ Like other nitroarene dioxygenases, 2NTDO catalyzes the dioxygenation of the aromatic moiety to *cis*-dihydroxylated intermediates that spontaneously form catecholic products and NO_2_^–^ (Scheme 2). To a lesser extent, the methyl group of nitrotoluene undergoes monooxygenation forming nitrobenzylalcohols.

Figure 1a shows substrate consumption and product formation for 2-nitrotoluene at different extents of turnover according to the concentration of NADH provided. 2-Nitrotoluene is transformed almost exclusively to 3-methylcatechol and equivalent amounts of NO_2_^–^ with the generation of only minor traces of 2-nitrobenzylalcohol. The mass balance of organic substrate and products confirms that 2NTDO carried out the two hypothesized hydroxylation reactions. The stoichiometric coefficients of substrate loss and product formation normalized to the amounts of NADH added, υ_*j*_, from Tables 1 and S7 allow for an assessment of the oxygenation efficiency of 2NTDO with 2-nitrobenzene. The O_2_ consumption coefficient, υ_O_2__, of 0.63 ± 0.01 mol/mol of NADH illustrates that some reduction equivalents of NADH were not involved in O_2_ activation by 2NTDO in this experiment series (section S4.3 and Table S4). 3-Methylcatechol and 2-nitrobenzylalcohol were formed at 0.62 ± 0.02 and 0.03 ± 0.01 mol/mol NADH, respectively. Detection of both dioxygenation products, 3-methylcatechol and NO_2_^–^, at equal stoichiometries (υ_NO_2_^–^_ = 0.63 ± 0.06) confirmed the accuracy of our analytical procedures and thus allowed for quantifying the dioxygenation reactions in Table 1 on the basis of NO_2_^–^ measurements.^61,65^ The stoichiometric coefficient for O_2_ consumption is identical within uncertainty, implying that all activated O_2_ is used in hydroxylation reactions. Accordingly, we did not observe any O_2_ uncoupling (*f*_O_2_-uc_ = 0.02 ± 0.03, Table 1, entry 2).

**Table 1:** Stoichiometries for O_2_ Activation and Dioxygenation of Substituted Nitroaromatic Substrates by 2NTDO and NBDO as well as the ^13^C-KIE and ^18^O-KIE Values of the Substrates^a^

entry	(co)substrate	υ_*j*_^b^	*f*_O_2_-uc_^c^	^18^O-KIE	^13^C-KIE
*2NTDO*					
1a	nitrobenzene	0.50 ± 0.02	0.33 ± 0.02		1.007 ± 0.001
1b	O_2_ (NB)	0.65 ± 0.01^d^		1.015 ± 0.001	
2a	2-nitrotoluene	0.62 ± 0.02	0.02 ± 0.03		1.006 ± 0.002
2b	O_2_ (2-NT)	0.63 ± 0.01^d^		1.016 ± 0.002	
3a	3-nitrotoluene	0.16 ± 0.02	0.84 ± 0.03		1.004 ± 0.001
3b	O_2_ (3-NT)	0.99 ± 0.01		1.018 ± 0.001	
4a	4-nitrotoluene	0.05 ± 0.01	0.94 ± 0.01		1.003 ± 0.001^e^
4b	O_2_ (4-NT)	0.85 ± 0.01		1.021 ± 0.003	
5a	2-fluoronitrobenzene	0.40 ± 0.02	0.36 ± 0.03		1.002 ± 0.004
5b	O_2_ (2-F-NB)	0.68 ± 0.01^d^		1.015 ± 0.001	
6a	3-fluoronitrobenzene	0.44 ± 0.03	0.35 ± 0.07		1.011 ± 0.006
6b	O_2_ (3-F-NB)	0.62 ± 0.01^d^		1.016 ± 0.001	
7a	4-fluoronitrobenzene	0.13 ± 0.01	0.83 ± 0.01		1.005 ± 0.001
7b	O_2_ (4-F-NB)	0.79 ± 0.01		1.019 ± 0.001	
8a	2-chloronitrobenzene	0.66 ± 0.05	0.21 ± 0.05		0.998 ± 0.002
8b	O_2_ (2-Cl-NB)	0.79 ± 0.01^d^		1.015 ± 0.001	
9a	3-chloronitrobenzene	0.10 ± 0.01	0.79 ± 0.02		1.011 ± 0.001
9b	O_2_ (3-Cl-NB)	0.51 ± 0.01^d^		1.016 ± 0.001	
10a	4-chloronitrobenzene	0.04 ± 0.01	0.92 ± 0.01		1.007 ± 0.006
10b	O_2_ (4-Cl-NB)	0.59 ± 0.01		1.013 ± 0.001	
11a	2-nitrophenol	0.07 ± 0.01	0.94 ± 0.01		1.000^f ^
11b	O_2_ (2-NP)	1.09 ± 0.01^d^		1.014 ± 0.001	
12	O_2_ (3-nitrophenol)	1.07 ± 0.01	1.00^g^	1.015 ± 0.001	
13a	4-nitrophenol	0.04 ± 0.01	0.94 ± 0.01		1.000^f ^
13b	O_2_ (4-NP)	0.80 ± 0.01		1.016 ± 0.001	
*NBDO*					
14a	2-nitrotoluene	0.18 ± 0.02	0.62 ± 0.01		1.018 ± 0.001^h^
14b	O_2_ (2-NT)	0.89 ± 0.01		1.018 ± 0.001	
15a	4-nitrotoluene	0.18 ± 0.02	0.74 ± 0.01		1.010 ± 0.001^h^
15b	O_2_ (4-NT)	0.80 ± 0.01		1.013 ± 0.001	

a Uncertainties correspond to 95% confidence intervals.

b NADH-normalized stoichiometry of (co)substrate consumption calculated with eq 1; substrate dihydroxylation is quantified on the basis of measured NO_2_^–^ concentrations.

c O_2_ uncoupling determined with eq 2.

d Without O_2_ background consumption according to eq S2.

e Reproduced from Pati et al.^40^ due to low turnover; see section S3.4.

f ^13^C-KIE set to unity; see section S3.4.

g No NO_2_^–^ detected.

h reproduced from Pati et al.^40^ excluding monooxygenation with kinetic model.^60^

In contrast to the case of 2-nitrotoluene, 2NTDO hydroxylated other substrates very inefficiently. Figure 1b shows the results of a substrate turnover experiment for 4-chloronitrobenzene. Coefficients for substrate consumption, υ_S_, and dioxygenation, υ_NO_2_^–^_, are small and identical at 0.04 mol/mol NADH, whereas O_2_ consumption is substantially higher (υ_*O*_2__ = 0.59 ± 0.01 mol/mol NADH, Table 1, entries 10a/b). Thus, only 8% of O_2_ consumption was utilized for substrate hydroxylation, whereas the remaining 92% led to unproductive O_2_ activation. We recovered up to 43% of the consumed O_2_ as H_2_O_2_ in additional assays (Table S6), confirming not only that a large fraction of the uncoupled O_2_ was present as ROS but also that these species were released into solution. The comparison of these data for 2-nitrotoluene and 4-chloronitrobenzene furthermore shows that the efficiency of oxygenation vs O_2_ uncoupling is highly variable.

**Figure 2:**
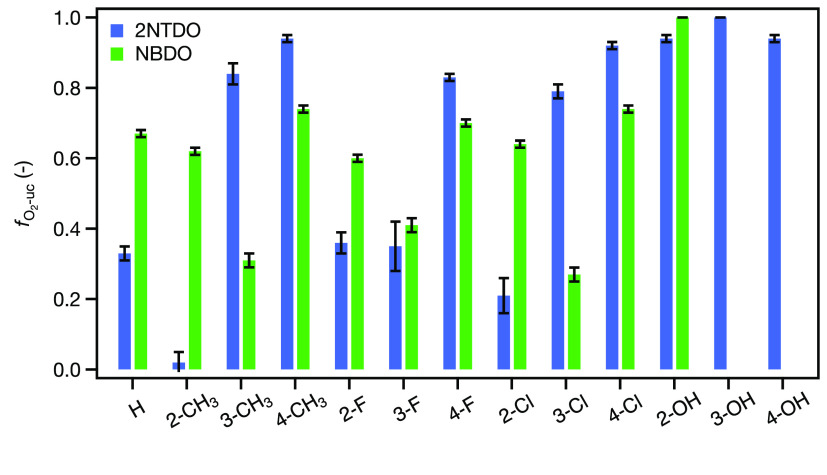
Extent of O_2_ uncoupling in 2-nitrotoluene dioxygenase (blue, 2NTDO) and nitrobenzene dioxygenase (green, NBDO^65^) with substituted nitrobenzenes (data from Table 1).

We systematically evaluated this substrate dependence of O_2_ uncoupling by 2NTDO for a broad range of structurally related compounds. All nitroaromatic substrates led to O_2_ consumption that exceeded the background O_2_ disappearance at 3 μM min^–1^ by at least 3-fold (Figures S1 and S5) whereas non-nitrated compounds, such as benzene or toluene, did not cause any O_2_ disappearance beyond the background rate (section S4.1). Figure 2 shows *f*_O_2_–uc_ values for nitrobenzene as well as methylated, fluorinated, chlorinated, and hydroxylated nitrobenzenes used as model compounds to study the effects of substrate molecular structure on nitroarene activities. Many of these compounds are known environmental contaminants that can undergo oxidative biodegradation.^85−89^ With exception of 2-nitrotoluene, all substrates lead to substantial O_2_ uncoupling and this unproductive path of O_2_ activation even predominated enzymatic activity. The type of aromatic substituent is largely irrelevant for the extent of hydroxylation vs O_2_ uncoupling. In assays containing chlorinated nitrobenzene, for example, *f*_O_2_-uc_ ranged from 20% to 90% (entries 8–10, Table 1). Nitrophenols exclusively promoted unproductive O_2_ activation (*f*_O_2_-uc_ > 0.9).

Figure 2 also shows the O_2_ uncoupling activity of NBDO with data from Pati et al.^65^ Compared to 2NTDO, *f*_O_2_-uc_ values for NBDO were confined to a smaller range of values between 0.31 ± 0.02 (3-nitrotoluene) and 0.74 ± 0.01 (4-chloronitrobenzene). Nitrophenol was not hydroxlyated by NBDO, similarly to what was found for 2NTDO. NBDO and 2NTDO also show very distinct substrate specificity. 2-Chloronitrobenzene, for example, differs in *f*_O_2_-uc_ values by 43% between assays of 2NTDO vs NBDO. Only one substrate, 3-fluoronitrobenzene, exhibited the extent of O_2_ uncoupling within <10% for both NBDO and 2NTDO. It is interesting to note that the eponymous and thus potentially optimized substrate for dioxygenation by 2NTDO, 2-nitrotoluene, lacks O_2_ uncoupling whereas NBDO shows a poor oxygenation efficiency with nitrobenzene as substrate (*f*_O_2_-uc_ = 0.67 ± 0.01). A more detailed discussion of the substrate-specific impacts on *f*_O_2_-uc_ values follows below.

### O_2_ Uncoupling in the Catalytic Cycle of Nitroarene Dioxygenases

**Scheme 3:**
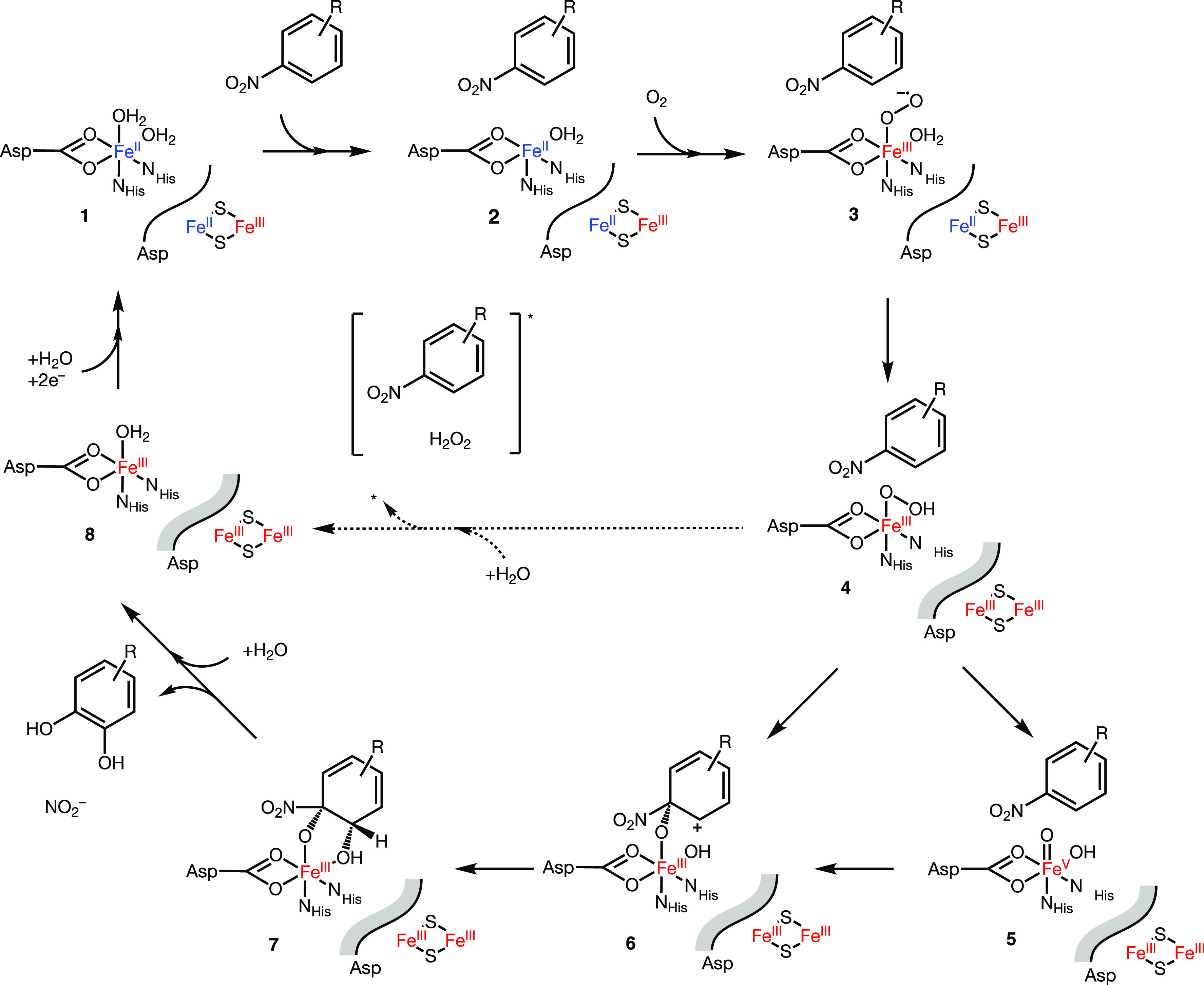
Catalytic Cycle of the Dioxygenation of Nitroaromatic Substrates by 2NTDO and NBDO Based on Studies of NDO and NBDO^39,65^ Illustration shows the non-heme Fe^II^ active site, a generic nitroaromatic substrate, and the [2Fe-2S] Rieske cluster in different oxidation states.

We analyzed the catalytic cycle of nitroarene dioxygenases outlined in Scheme 3 for possible O_2_ uncoupling reactions by dissecting the rate-limiting steps leading to the consumption of O_2_ and the aromatic substrate. To that end, we quantified ^18^O-KIEs for O_2_ activation in Fe-oxygen species according to the methodology applied previously to study O_2_ activating processes in non-heme ferrous iron oxygenases.^65,67,68,70−72,90^^13^C-KIEs were used to characterize the timing of substrate hydroxylation. The corresponding data are compiled in Table 1.

#### Rate-Limiting Steps of O_2_ Activation

**Figure 3:**
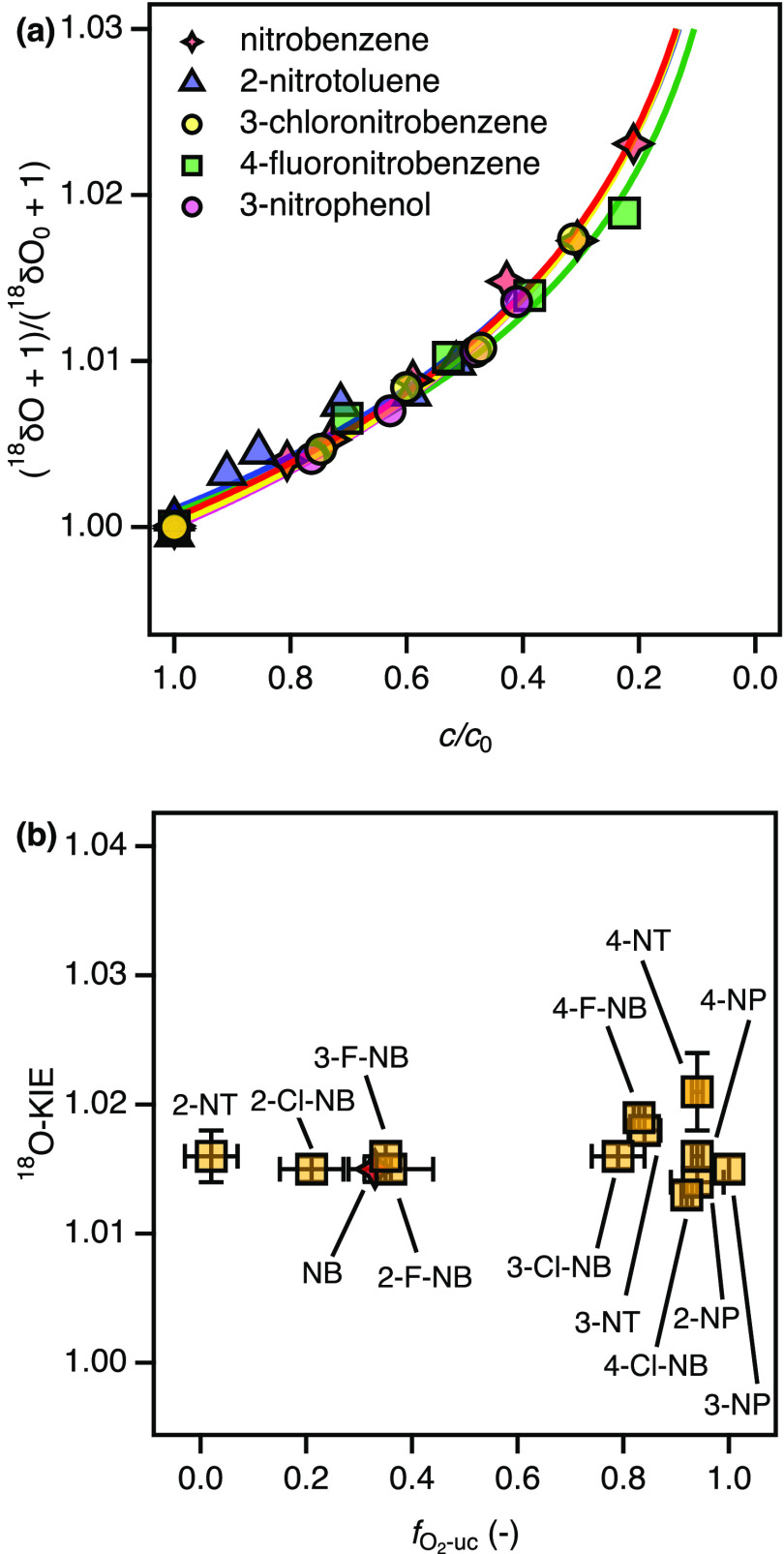
Changes of ^18^O/^16^O ratios (a) and ^18^O-KIE of O_2_ activation (b) by 2NTDO in the presence of various substrates.

We derived the ^18^O-KIEs of O_2_ by 2NTDO for the entire set of nitroaromatic substrates by evaluating changes in ^18^O/^16^O ratios of the residual dissolved O_2_ at different extents of turnover (Figure 3a). We observed moderately large O isotope fractionation which followed the trends described in eq 5. All ^18^O-KIEs were confined to values between 1.013 and 1.020 (Table 1) with an average value of 1.016, and they are thus independent of the elementary reaction step leading to O_2_ uncoupling (Figure 3b). This observation strongly suggests the formation of one type of Fe-oxygen species regardless of the nitroaromatic substrate. Comparison of experimental ^18^O-KIE values with theoretical ^18^O equilibrium isotope effects (^18^O-EIEs) of Mirica et al.^68^ imply the formation of ferric iron (hydro)peroxo species (Fe^III^–OO(H), ^18^O-EIE of 1.0172), a species that has previously been postulated to catalyze oxygenations by naphthalene dioxygenase.^31,37^ Smaller ^18^O-KIE values, by contrast, stand for Fe-superoxo species (^18^O-EIE of 1.0080), whereas higher ^18^O-KIE have been assigned to Fe^IV^=O (^18^O-EIE of 1.0287).^68^

The observation of a narrowly confined ^18^O-KIE for O_2_ activation by 2NTDO is consistent with data obtained for NBDO^65^ and suggests that the two nitroarene dioxygenases follow the same initial catalytic mechanism. As shown in Scheme 3 in reactions **1** → **2** → **3** → **4**, the presence of substrate in the active site induces the loss of a H_2_O ligand at the non-heme Fe (**2**) followed by O_2_ binding and activation (**3**). Substrate binding ultimately promotes the electron transfer from the Rieske cluster (Fe^II^–Fe^III^ → Fe^III^–Fe^III^ in **3** → **4**) that enables generation of the ferric Fe-(hydro)peroxo species (**4**) in the rate-limiting step of O_2_ consumption. A common mechanism of O_2_ activation in nitroarenes confirms the widely made observation that the kinetics of O_2_ activation are triggered by the substrate but do not involve interactions of the substrate with the non-heme Fe species.^35^ It follows from the conserved ^18^O-KIE values that the substantial substrate-dependence of O_2_ uncoupling must originate from reaction steps after generation of species **4**.

A number of observations suggest that O_2_ uncoupling would happen primarily from species **4**. Previous works with NBDO have shown that the first step of the asynchronous hydroxylation of the substrate (**4** → **5** or **4** → **6**) is irreversible.^38,40^ O_2_ uncoupling therefore has to occur from **4** or **5**. This conclusion is supported by the fact that the substrate has to be released in an unreacted form, in agreement with the mass balances of aromatic compounds illustrated above (Figure 1). Finally, we detect a large share of the uncoupled O_2_ as H_2_O_2_ in the assay solutions. As shown in Table S6, H_2_O_2_ concentrations do not account for all of the uncoupled O_2_, suggesting that some H_2_O_2_ could have reacted further with electron rich moieties within the proteins or the buffer. We rule out a release of superoxide from species **3** given that this process would need to occur reversibly to be consistent with the ^18^O-KIEs. O_2_ uncoupling from species **5**, on the other hand, is an unlikely source of H_2_O_2_ because the cleavage of O–O bonds is typically irreversible.^70^ The most likely reaction of **5** with concomitant loss of O_2_ is a monooxygenation reaction with nitrotoluene substrates in which the release of reduced oxygen would occur as H_2_O.^65^

#### Timing of Substrate Hydroxylation

**Figure 4:**
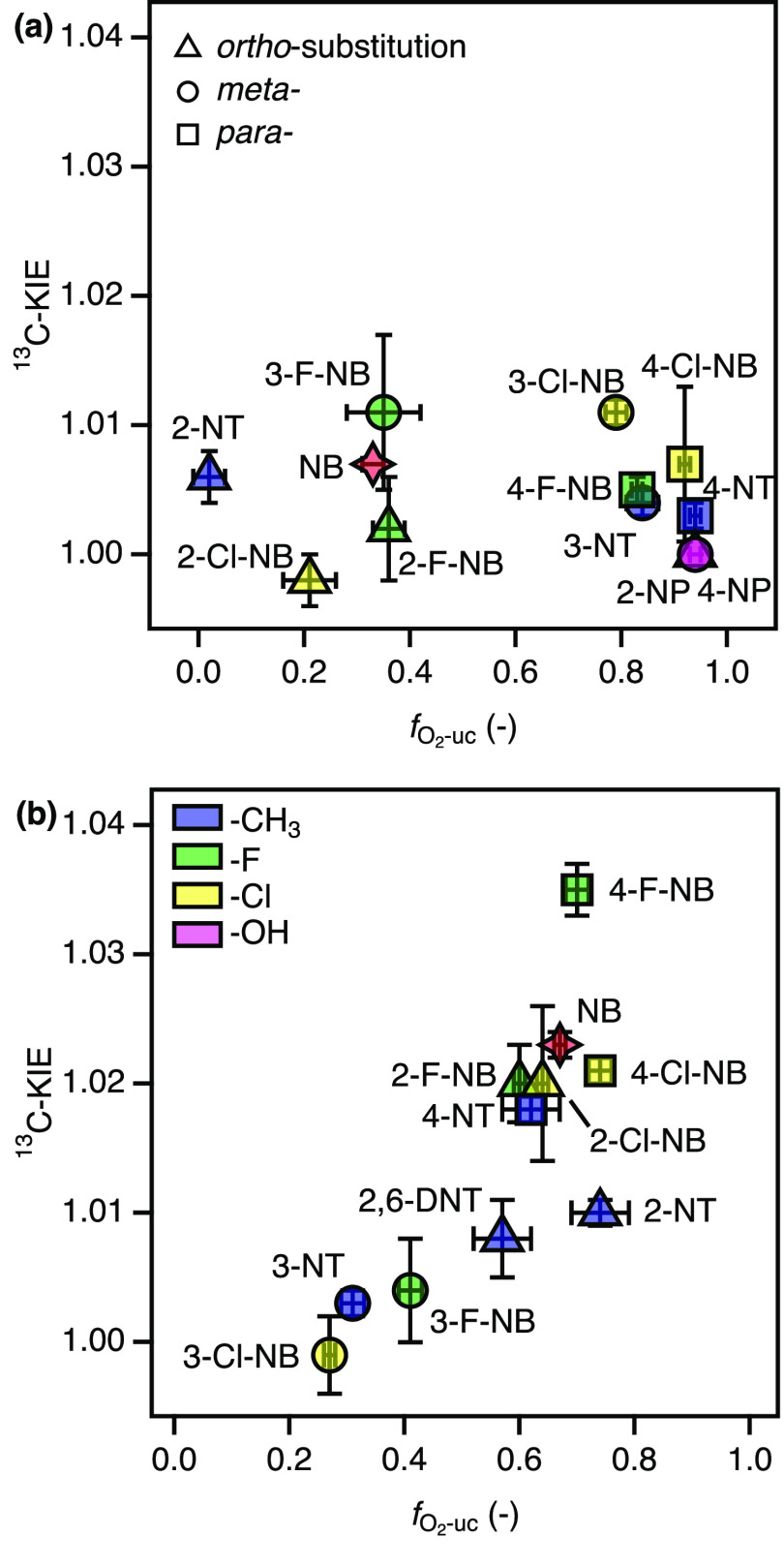
^13^C-KIEs of substrate dioxygenation by 2NTDO (a) and NBDO (b) vs fraction of uncoupled O_2_ activation, *f*_O_2_-uc_. Panel (b) was constructed with data from Pati et al.^65^ and this study.

The ^13^C-KIE values in the 12 reactive substrates were derived from the C isotope fractionation as shown in Figure S7 on the basis of eqs 3 and 4. Note that due to the low turnover of many substrates, their carbon isotope fractionation is difficult to detect (see discussion in section S3.4). All ^13^C-KIE values are small, vary between unity and 1.01 (Table 1), and are not correlated with O_2_ uncoupling as shown in Figure 4a. These values are notably smaller than experimentally observed and theoretically derived intrinsic ^13^C-KIEs which can be as large as 1.024 and 1.039, respectively.^40,86^ The observation of small isotope fractionation after the rate-limiting step of the catalytic cycle (i.e., O_2_ activation) is nevertheless counterintuitive. Such kinetic mechanisms typically show a complete absence of substrate isotope fractionation as shown for flavin-dependent oxygenases.^74^ We posit that the observed C isotope fractionation and the nonunity of ^13^C-KIEs associated with the activity of 2NTDO are due to the O_2_ uncoupling process and reflect the reaction path **4** → **5** → **6**. This path is also distinct from the one postulated previously for NBDO.^65^ To observe C isotope fractionation in the unreacted substrate released through uncoupling from species **4**, the following reactions would need to involve isotope-sensitive bonding changes and be reversible. While hydroxylations of aromatic carbon in reaction **5** → **6** fulfils the first requirement with a large intrinsic ^13^C-KIE for the formation of the Fe^V^-(oxo)hydroxo species,^40^ reaction **4** → **5** is presumably not reversible for reasons outlined above. To that end, C isotope fractionation from the hydroxylation does not alter the ^13^C/^12^C ratio of the nitroaromatic substrate in species **4** that could be observed upon O_2_ uncoupling. Indirect confirmation for this interpretation comes from comparison of the identical type of data for NBDO in Figure 4b.^65^ In this case, the progressive expression of a ^13^C-KIE with increasing *f*_O_2_-uc_ values is due to a partly reversible reaction **4** → **6** which alters the ^13^C/^12^C ratio of the remaining substrate. The substrate C isotope fractionation observed therefore increases with increasing extent of O_2_ uncoupling.

### Effect of Substrate Structure and Active Site Residues on O_2_ Uncoupling

**Figure 5:**
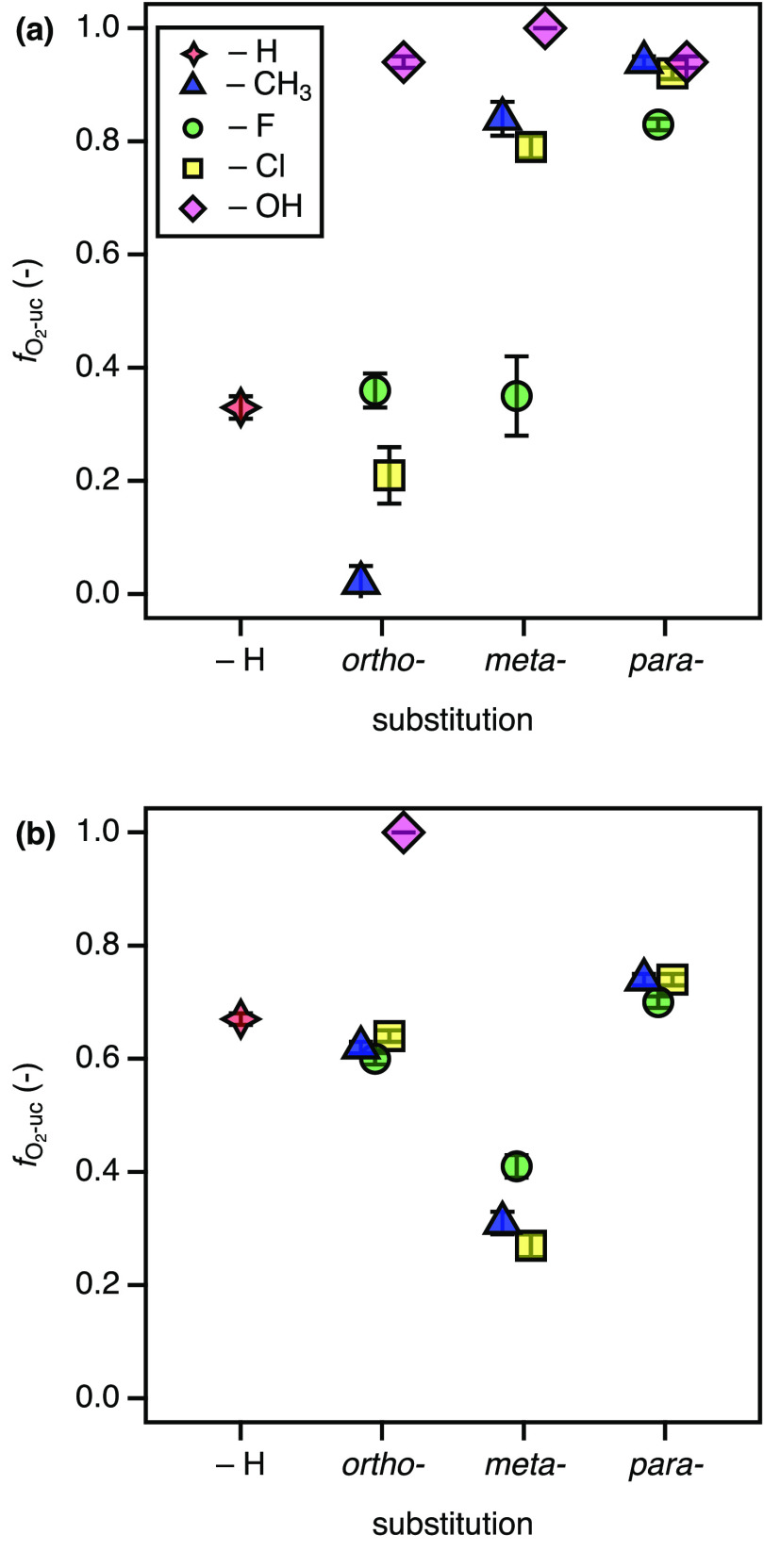
Extent of O_2_ uncoupling, *f*_O_2_-uc_, caused by different substituted nitrobenzenes in 2NTDO (a) and NBDO (b) vs position of the aromatic substituent of the substrate. The legend in panel (a) applies to both figures.

We evaluated the consequences of structural factors pertinent to substrate substituent types and positions as well as the enzyme’s active site to elucidate possible causes for the distinct substrate specificity and O_2_ uncoupling behavior shown in Figure 5. 2NTDO and NBDO share 95% sequence identity and differ only slightly in their active site residues.^64^ While both enzymes exhibit the Asn258 residue responsible for H-bonding to the oxygen atoms of the nitro group, 2NTDO hosts an Ile residue at position 293 where NBDO has a more bulky Phe. This additional space in the active site of 2NTDO was hypothesized to allow for a favorable binding of 2-nitrotoluene so that the aromatic ring is oriented toward the reactive Fe-oxygen species for dioxygenation despite its *ortho*-methyl substituent.^64^ In fact, we observed a reduced O_2_ uncoupling for 2NTDO with 2-nitrotoluene and other *ortho*-substituted substrates (Figure 5a). Nitrophenol substrates are not discussed further because these compounds are not dioxygenated by any of the two enzymes. Based on this reasoning, the increased *f*_O_2_-uc_ values for chlorine and methyl substituents in *meta*- and any substituent in *para*-position can be explained by a poor substrate fit in the active site as primary origin of O_2_ uncoupling. This interpretation is also supported qualitatively by the relatively lower *f*_O_2_-uc_ values for nitrobenzene and, given the smaller size of fluorine, for 3-fluorobenzene.

We observed distinct trends for *f*_O_2_-uc_ values in NBDO (Figure 5b). Here, the eponymous substrate nitrobenzene exhibits a relatively high extent of O_2_ uncoupling of about 60% which is also found for *ortho*- and *para*-substituted nitrobenzenes. By contrast, *meta*-substitution with −CH_3_, −F, and −Cl allowed for a more efficient dioxygenation of the substrates. The finding that *f*_O_2_-uc_ values for the methyl-, fluoro-, and chloro-substituted nitrobenzenes with NBDO cluster together reinforces the interpretation of data for 2NTDO that the structure of the substrate is a likely determinant of O_2_ uncoupling. At first sight, electronic effects appear to be of negligible relevance even though −CH_3_ vs halogen substituents alter the partial atomic charges of the C atoms and thus the susceptibility for attack by electrophilic Fe-oxygen species in RDOs.^32^

None of the trends revealed in Figure 5, however, allows one to rationalize the preference of 2NTDO and NBDO for oxygenation of *ortho*- and *meta*-substituted nitrobenzenes, respectively, or the considerable magnitude of O_2_ uncoupling by both enzymes. A hypothesis proposed for the uncoupled O_2_ activation vs substrate monooxygenation by α-ketoglutarate dependent non-heme ferrous iron oxygenases,^47^ an enzyme class that uses a different mechanism for O_2_ activation than RDOs,^18,20−22^ suggests that the lifetime of reactive Fe-oxygen species is one of the crucial factors. An extended lifetime of the Fe(IV)-oxo intermediate, for example, due to the presence of substrates reacting more slowly through electrophilic oxygen addition, could lead to uncoupled O_2_ activation, as compared to more reactive substrates. No such trends are apparent in our data for 2NTBO and NBDO. Even though nitrotoluenes could be considered better substrates for electrophilic attack of Fe-oxygen species in 2NTDO and NBDO, they show *f*_O_2_-uc_ values identical to those of chlorinated and fluorinated nitrobenzenes. Instead, we hypothesize that the electronic properties of the substrate bound in the active site pocket exert some allosteric control of O_2_ activation and could thus also be responsible for the efficiency of hydroxylation. We found recently for another RDO (naphthalene dioxygenase^35^) that the electron affinity of the substrate bound in the active site modulates the thermodynamics of the metal-to-substrate charge transfer from the Rieske cluster through the H_2_O ligand in reaction **1** → **2** (Scheme 3). Given that the presence of the substrate is also accompanied by conformational changes in the active site that allow for O_2_ binding at the non-heme Fe, we speculate that these processes result in an orientation of the substrate toward reactive Fe-oxygen species that is less likely to undergo O_2_ uncoupling. Further theoretical studies on nitroarene dioxygenases are warranted to examine this hypothesis.

## Environmental Significance

The observation of substantial O_2_ uncoupling in almost all enzyme–substrate combinations investigated in our study suggests that the unproductive activation of O_2_ is an important and largely overlooked path in the catalysis of contaminant oxygenation by nitroarene dioxygenases. Given that RDOs all share the catalytic mechanisms in which O_2_ activation to reactive Fe-oxygen species occurs without interactions with the substrate,^18,20−22,35^ we posit that O_2_ uncoupling is likely an abundant phenomenon among RDOs. O_2_ uncoupling is thus of relevance for many, if not most, contaminant dioxygenation pathways.^10,91^ The relative extent of O_2_ uncoupling observed among different substituted nitrobenzenes used as model substrates for the two nitroarene dioxygenases, however, is difficult to rationalize in terms of active site properties and simple structural and electronic descriptors of the substrates. Molecular structures of potential RDO substrates that would appear to favor dioxygenation may or may not be accompanied by O_2_ uncoupling. The ambiguity of identifying productive enzyme–substrate combinations not only makes it very difficult to assess or even predict oxidative biodegradation in structure–reactivity relationships but also could challenge the interpretation of correlations of enzyme activity with productive contaminant transformation.^92^

The release of unreacted substrate during the O_2_ uncoupling steps of the catalytic cycle of RDOs also has severe consequences for the assessment of the extent of contaminant transformation from changes of the isotopic composition in the remaining contaminant by compound-specific isotope analysis (CSIA).^93,94^ Many applications of CSIA have demonstrated successfully that enzymatic catalysis of contaminant transformation can be tracked by the substrate isotope fractionation that arises from kinetic isotope effects of bond cleavage reactions. Unfortunately, the substrate-dependent occurrence of O_2_ uncoupling modulates the extent of observable substrate isotope fractionation from isotope effects of aromatic compound hydroxylations by RDOs in an unpredictable way. This phenomenon likely precludes the quantitative interpretation of isotope fractionation associated with the dioxygenation processes. Our insights would therefore call for a re-evaluation of stable isotope based data from biodegradation reactions of various contaminants that are likely catalyzed through oxygenations by non-heme iron oxygenases^95−101^ once the O_2_ uncoupling behavior of the involved enzymes is known.

Finally, the quantitative evaluation of O_2_ uncoupling reactions in enzyme assays presented in our study offers new avenues to study the hypothesis of ROS-driven adaptation of the RDO substrate spectrum toward new substances.^55−57^ Besides having a potentially detrimental effect on RDO activity through enzyme self-hydroxylation^41^ and redirecting metabolic fluxes to sustain defense mechanisms,^48^ ROS generated from O_2_ uncoupling have been postulated to increase mutation rate and selective pressure that lead to an accelerated adaptation of RDOs to xenobiotic compounds. In fact, 2NTDO and NBDO studied here originate from single isolated bacteria that might not necessarily represent the best or most common versions of the enzymes. Under laboratory conditions, shifts of substrate specificity of RDOs can occur within relatively short time scales of weeks to months^76,102^ and they have been accompanied by mutations of selected amino acid residues unrelated to the enzymes’ active site. Given that O_2_ uncoupling and generation of ROS is potentially one of the first biochemical responses to exposure to new or alternate substrates, an evaluation of *f*_O_2_-uc_ values for RDOs with different degrees of adaptation to new substrates are needed. Such works would also allow further evaluation of the current substrate specificities of 2NTDO and NBDO as a possible evolutionary compromise to minimize oxidative stress triggered by the continuous exposure to mixtures of structurally similar contaminants in the environment.

## Supporting Information Available

Chemicals and biological materials used, detailed method descriptions for protein purification, enzyme assays, chemical, and isotopic analyses, additional results on enzyme kinetics, reaction stoichiometries, and carbon and oxygen isotope fractionation (PDF)
