# Supplementary material for: Elucidating the Role of O2 Uncoupling in the Oxidative Biodegradation of Organic Contaminants by Rieske Non-heme Iron Dioxygenases
Source: ACS Environ Au. 2022 Jul 7;2(5):428–40. doi: 10.1021/acsenvironau.2c00023 (PMC9502038; doi:10.1021/acsenvironau.2c00023)
Supplement: Supplementary file 1 — vg2c00023_si_001.pdf [file vg2c00023_si_001.pdf]

Supporting Information for

Elucidating the Role of O_2 Uncoupling in the Oxidative Biodegradation of Organic Contaminants by Rieske Non-Heme Iron Dioxygenases

Charlotte E. Bopp,[1,2] Nora M. Bernet,[1] Hans-Peter E. Kohler,[1] and Thomas B. Hofstetter*[1,2]

[1]Eawag, Swiss Federal Institute of Aquatic Science and Technology, CH-8600 Dübendorf, Switzerland, [2]Institute of Biogeochemistry and Pollutant Dynamics (IBP), ETH Zürich, CH-8092 Zürich, Switzerland

*Corresponding author: thomas.hofstetter@eawag.ch
phone +41 58 765 50 76, fax +41 58 765 50 28

21 Pages, 7 Figures, 8 Tables

Contents

S1 Chemicals and Biological Materials **3**

S2 Purification of nitroarene dioxygenases **3**
S2.1 Bacterial strains and growth conditions . . . 3
S2.2 Purification of ferredoxin . . . 4
S2.3 Purification of reductase . . . 4
S2.4 Purification of 2NTDO and NBDO . . . 6

S3 Experimental and Analytical Procedures **7**
S3.1 Enzyme assay . . . 7
S3.1.1 Control experiments . . . 7
S3.1.2 Quantification of O_2 uncoupling and background consumption . . . 8
S3.2 Chemical analyses . . . 12
S3.2.1 HPLC-based quantification of organic compounds . . . 12
S3.2.2 Quantification of dissolved O_2 . . . 13
S3.2.3 Quantification of NO_2^- in enzyme assays . . . 13
S3.3 Enzyme Kinetics . . . 13
S3.4 $^{13}C/^{12}C$ ratio analysis in substrates with limited turnover . . . 14

S4 Additional Results **15**
S4.1 Survey of initial rates of O_2 consumption rates . . . 15
S4.2 H_2O_2 quantification . . . 17
S4.3 Stoichiometric coefficients of substrate consumption and product formation 18
S4.4 Oxygen isotope fractionation of dissolved O_2 . . . 19
S4.5 Carbon isotope fractionation nitroaromatic substrate hydroxylation . . . 19
S4.6 Enzyme Kinetics . . . 20

S1 Chemicals and Biological Materials

The materials used in this work is largely identical to our previous study by Pati et al.[1] and this information is reproduced here with minor modifications.

The following were used as substrates in enzyme assays and used as received. From Sigma-Aldrich (Buchs SG, Switzerland) or Merck (Schaffhausen, Switzerland), we purchased nitrobenzene (99%), 2-nitrotoluene (99%), 3-nitrotoluene (99%), 2-, 3-, 4-chloronitrobenzene (99%), 2-, 3-, 4-fluoronitrobenzene (99%), 2-nitrophenol (99%), 2,6-dinitrotoluene (98%), 2,4-dinitrotoluene (98%), benzoic acid (99%). 4-nitrotoluene (98%) was purchased from Fluka. Catechol (99%), 3- and 4-methylcatechol (95%), 3- and 4-chlorocatechol, 3- and 4-fluorocatechol, 2-, 3-, and 4-nitrobenzylalcohols, and sodium nitrite ($NaNO_2$, 99%) were used as reference compounds to quantify reaction products. 4-Morpholine-ethanesulfonic acid monohydrate (MES, 99%) and potassium phosphate monobasic (KH_2PO_4, 99.5%) were used as buffers and pH was adjusted with sodium hydroxide (NaOH, 99%) and hydrochloric acid (HCl, 32 %). In addition, β-nicotinamide adenine dinucleotide reduced disodium salt (NADH, 97%), peroxidase from horseradish (type VI, 250 pyrogallol units/mg), hydrogen peroxide (H_2O_2, 30%), 4-methoxyaniline (99%), Ampliflu (98%) and ammonium ferrous sulfate hexahydrate ($(NH_4)_2Fe(SO_4)_2$, 99%) were used in enzyme assays. *N*-(1-naphthyl)ethylenediamine (NED, 99%), sulfanilamide (99%), and sodium sulfite anhydrous ($NaSO_3$, 97%) were used for quantitative analyses of NO_2^-. Methanol (LC/MS grade, 99.99%) was purchased from Fisher Scientific (Reinach, Switzerland). He (99.999%), N_2 (99.999%), and O_2 (99.9995%) gases were from Carbagas (Rümlang, Switzerland). Aqueous solutions were prepared in nanopure water (18.2 MΩ· cm, Barnstead NANOpure Diamond Water Purification System). BTGED buffer containing 50 mM Bis-Tris (Sigma-Aldrich, 98%), 5% v/v glycerol (Sigma-Aldrich, 99%), 5% v/v ethanol (Merck, 99.9%), and 1 mM dithiothreitol (Fisher Scientific, 99%) was used for protein purification.

S2 Purification of nitroarene dioxygenases

Bacterial growth conditions and purification procedures were largely adapted from previous works.[2–4] In the following, we describe the complete purification protocol for the four enzyme components reductase, ferredoxin, 2-nitrotoluene dioxygense (2NTDO) and nitrobenzene dioxygenase (NBDO) adapted for this study. Chromatographic separations were performed on an automated fast protein liquid chromatography system (ÄKTA FPLC, GE Healthcare Life Science) with columns manually packed with resins from Cytiva (Marlborough, USA).

S2.1 Bacterial strains and growth conditions

Escherichia coli DH5α(pDTG800)[2] carrying the *ntdAaAbAcAd* genes from *Acidovorax* sp. strain JS42 was used for purification of the terminal oxygenase component of 2NTDO. The strain was grown at 37°C to late exponential phase (OD_{600} = 3-4) with shaking in LB (5 g L^{-1} yeast extract, 10 g L^{-1} tryptone, 5 g L^{-1} NaCl) containing 200 µg mL^{-1} ampicillin before 150 µM isopropyl-β-D-thiogalactopyranoside (IPTG) was added followed. Thereafter the incubation proceeded for three additional hours at 28°C.

E. coli VJS415(pK19::927)[3] carrying the *nbzAaAbAcAd* genes form *Comomonas* sp. strain JS765[5] was used for purification of the terminal oxygenase component of NBDO. *E. coli* DH5α(pJPK13::Fd)[3], which carries only the *nbzAb* gene, was used for the isolation of the ferredoxin component that is identical for the 2NTDO and NBDO enzyme systems.[2] Both strains were grown at 37°C with shaking in LB containing 100 μg L^{-1} kanamycin sulfate to late exponential phase without induction by IPTG.[4] *E. coli* BL21(DE3)(pDTG871) carrying only the *nbzAa* gene was used for isolation of the reductase component identical for the 2NTDO and NBDO enzyme systems.[2] The strain was grown at 37°C with shaking in terrific broth (24 g L^{-1} yeast extract, 12 g L^{-1} tryptone, 4 g L^{-1} glycerol, 2.3 g L^{-1} KH_2PO_4, 16.4 g L^{-1} K_2HPO_4) containing 200 µg mL^{-1} ampicillin. When the cultures reached late exponential phase (OD_{600} = 4-5), 100 μM IPTG were added and cells were incubated for additional three hours at 30°C. All cells were harvested by centrifugation (6'000 rpm at 4°C for 20 min), redissolved at 1 g mL^{-1} in BTGED buffer (50 mM Bis-Tris, 5% glycerol, 5% ethanol, 1 mM sodium dithiothreitol, pH 6.8) and stored at –20°C.

S2.2 Purification of ferredoxin

90 g of frozen cell pellet in BTGED buffer were thawed on ice and mixed with 1 mg mL^{-1} DNase I. The cell suspension was homogenised and passed twice through a chilled French pressure cell (1000 bar) and centrifuged at 10'000 *g* for 60 min at 4°C. The supernatant was passed through a 0.45 μm hydrophilic PVDF syringe filter and dialyzed in 2 L of fresh BTGED buffer and loaded onto a Q Sepharose XL ion exchange column (approximate bed volume of 200 mL) equilibrated with 600 mL BTGED. Unbound protein was removed with 200 mL BTGED at 2.5 mL min^{-1} followed by a linear gradient from 0 to 500 mM KCl in BTGED for 3 column volumes (CV). Fractions containing ferredoxin were identified by absoption at 456 nm[2] towards the end of the gradient and were combined and concentrated by ultrafiltration (10 kDa membrane, 1 bar nitrogen gas). Afterwards, ammonium sulfate was added to the concentrate to a final concentration of 1.5 M and the solution was loaded onto an octyl-sepharose 4 fast flow column (20 mL bed volume) equilibrated with 100 mL of 1.5 M $(NH_4)_2SO_4$ in BTGED. Unbound protein including ferredoxin was removed with 50 mL of 1.5 M $(NH_4)_2SO_4$ in BTGED at 1 mL min^{-1}. Fractions containing ferredoxin were pooled and concentrated by ultrafiltration. The concentrate was loaded onto a phenyl-sepharose 6 fast flow (high sub) column (20 mL bed volume) equilibrated with 100 mL 1.5 M $(NH_4)_2SO_4$ in BTGED. Again, ferredoxin eluted with the unbound protein which was removed with 200 mL of 1.5 M $(NH_4)_2SO_4$ in BTGED at 1 mL min^{-1}. Fractions containing ferredoxin were combined and concentrated by ultrafiltration. The buffer was exchanged to 50 mM MES (pH 6.8) and aliquots were stored at –80°C until needed. Table S1 gives an overview of the purification steps.[6]

S2.3 Purification of reductase

74 g of frozen cell pellet in BTGED buffer were thawed on ice and mixed with 1 mg mL^{-1} DNase I. The cell suspension was homogenised and passed twice through a chilled French pressure cell (1000 bar) and centrifuged at 10'000 *g* for 60 min at 4°C. The supernatant was passed through a 0.45 μm hydrophilic PVDF syringe filter and dialyzed in 2 L of fresh

Table S1 Purification of the enzyme components of the enzyme systems 2NTDO and NBDO.[6] Enzymes were purified repeatedly with similar results as shown in these examples.

Enzyme	step	total protein[a] (mg)	total activity (units)	specific activity[b] (units mg^{-1})	fold purification	yield (%)	purity[c] (%)	purity[d] %
ferredoxin	crude extract[e]	7275	464,700	64		100	9	
	Q-Sepharose	861	225,600	262	4.1	49	38	
	Octyl-sepharose	461	232,300	504	7.9	50	72	
	Phenyl-sepharose (hs)	178	124,200	697	10.9	27	100	95
reductase	crude extract[f]	4325	15,000	3.5		100	16	
	Q-Sepharose	561	3,250	5.8	1.7	22	27	
	Phenyl-sepharose (hs)	114	2,560	22.5	6.5	17	105	
	SEC[g]	97	2,090	21.5	6.2	14	100	51
NBDO	crude extract[h]	6213	42,800	7		100	2	
	Q-Sepharose	162	17,100	105	15.3	40	24	
	Phenyl-sepharose (hs)	34	15,000	438	63.5	35	100	92

[a] Protein concentration determined by Bradford assay using bovine serum albumin dilutions for calibration;
[b] based on activity tests according to Parales et al.[2] [c] based on specific activity;
[d] based on SDS-PAGE (10-20% Tricine gels, Invitrogen); [e] from 90 g of wet weight *E.coli* cell pellet (from 40 L of bacterial culture);
[f] from 74 g of wet weight *E.coli* cell pellet (from 20 L of bacterial culture);
[g] SEC did not markedly increase purity but removed O_2 consuming contamination; [h] from 68 g of wet weight *E.coli* cell pellet (from 65 L of bacterial culture).

BTGED buffer and loaded onto a Q Sepharose XL ion exchange column (approximate bed volume of 200 mL) equilibrated with 600 mL BTGED. Unbound protein was removed with 200 mL BTGED at 2.5 mL min^{-1} followed by a linear gradient from 0 to 500 mM KCl in BTGED for 3 CV. Fractions containing recuctase were identified by absoption at 460 nm and activity tests[2] in the middle of the gradient and were combined and concentrated by ultrafiltration (30 kDa membrane, 1 bar nitrogen gas). Afterwards, ammonium sulfate was added to the concentrate to a final concentration of 1 M and the solution was loaded onto phenyl-sepharose 6 fast flow (high sub) column (20 mL bed volume) equilibrated with 100 mL 1.5 M $(NH_4)_2SO_4$ in BTGED. Unbound protein was removed with 200 mL of 1 M $(NH_4)_2SO_4$ in BTGED at 1 mL min^{-1} followed by a 12 CV gradient to 0 mM $(NH_4)_2SO_4$ in BTGED. Fractions containing reductase eluted towards the end of the gradient and were pooled and concentrated by ultrafiltration. In contrast to previous procedures[2–4], the third chromatographic step was replaced by size exclusion chromatography (SEC) due to an O_2 consuming impurity (see section S3.1.1). Pooled sample of less than 3 mL was loaded onto a HiLoad Superdex 200 pg (120 mL bed volume, GE Healthcare Life Science) equilibrated with 200 mL of MES buffer (50 mM, pH6.8) and run isocratically at 1 mL min^{-1}. Fractions containing reductase were combined and concentrated by ultrafiltration and aliquots were stored at –80°C until needed. Table S1 gives an overview of the purification steps.[6]

S2.4 Purification of 2NTDO and NBDO

We performed identical steps for the purification of 2NTDO and NBDO as follows. 68 g of frozen cell pellet in BTGED buffer were thawed on ice and mixed with 1 mg mL^{-1} DNase I. The cell suspension was homogenised and passed twice through a chilled French pressure cell (1000 bar) and centrifuged at 10'000 *g* for 60 min at 4°C. The supernatant was passed through a 0.45 μm hydrophilic PVDF syringe filter and dialyzed in 2 L of fresh BTGED buffer and loaded onto a Q Sepharose XL ion exchange column (approximate bed volume of 200 mL) equilibrated with 600 mL BTGED. Unbound protein was removed with 200 mL BTGED at 2.5 mL min^{-1} followed by a linear gradient from 0 to 500 mM KCl in BTGED for 3 CV. Fractions containing oxygenase were identified by activity tests[2] in the beginning of the gradient and were combined and concentrated by ultrafiltration (100 kDa membrane, 1 bar nitrogen gas). Afterwards, ammonium sulfate was added to the concentrate to a final concentration of 1 M and the solution was loaded onto phenyl-sepharose 6 fast flow (high sub) column (20 mL bed volume) equilibrated with 100 mL 1.5 M $(NH_4)_2SO_4$ in BTGED. Unbound protein was removed with 200 mL of 1 M $(NH_4)_2SO_4$ in BTGED at 1 mL min^{-1} followed by a 12 CV gradient to 0 mM $(NH_4)_2SO_4$ in BTGED. Fractions containing oxygenase eluted after the end of the gradient and were pooled and concentrated by ultrafiltration. The buffer was exchanged to 50 mM MES (pH 6.8) and aliquots were stored at –80°C until needed. Table S1 gives an overview of the purification steps.[6]

S3 Experimental and Analytical Procedures

S3.1 Enzyme assay

S3.1.1 Control experiments

We characterized the O_2 background consumption systematically with a number of control experiments shown in Table S2. The assays were set up as controlled substrate turnover experiments (see main manuscript) with initial NADH concentrations of 250 μM. Figure S1 displays the O_2 consumption in the first 10 mins after NADH addition from which initial zero-order rates of O_2 consumption, ν_0, were determined in μM min $^{-1}$.

Assays without any of the three enzymes, reductase (red), ferredoxin (fer), and the oxygenase of 2NTDO (oxy), or NADH did not consume any O_2 ($|\nu_0| < 1$). Assay without substrate consumed O_2 at a small rate of 2.92 ± 0.09 μM min^{-1} (Fig. S1a). This rate is still significantly smaller than the target reaction rate in the enzyme assay (17.9 ± 0.4 μM min^{-1}). Controls with individual enzyme components (reductase, ferredoxin, and oxygenase) consumed less O_2 (Fig. S1b) suggesting that the O_2 consumption in the assay lacking substrate is not the result of an impurity but a reaction catalyzed by the full 2NTDO enzyme system. This is supported by the fact that no O_2 was consumed in assays without NADH. The modification of the reductase purification discussed in section S2.2 to include size exclusion chromatography (SEC), reduced the initial O_2 consumption rate from 1.24 ± 0.06 to 0.22 ± 0.09 μM min^{-1}. However, the O_2 consumption process evident in the "reductase only" samples does not appear to be as relevant when the whole multicomponent enzyme system is present as the impackt of the SEC purification was negligible in the "no substrate" samples (Table S2, entries 4 and 5).

Table S2 Control experiments for 2NTDO-catalyzed reactions performed for the assessment of background O_2 consumption in controlled substrate turnover experiments with nitrobenzene (NB).[a] Concentrations of assay components are indicated. Initial rates of O_2 consumption, ν_0, were derived from linear fits to the data points obtained within the first 2 minutes after addition of NADH.

entry	description	red 0.15 μM	fer 1.8 μM	oxy 0.15 μM	NB 100 μM	NADH 250 μM	ν_0[b] (μM min^{-1})
1	no NADH	✓[c]	✓	✓	✓	–	0.16±0.11
2	no NADH	✓[d]	✓	✓	✓[e]	–	-0.91±0.04
3	no enzyme	–	–	–	✓	✓	–1.11±0.05
4	no substrate	✓[c]	✓	✓	–	✓	2.92±0.09
5	no substrate	✓[d]	✓	✓	–	✓	2.86±0.11
6	reductase only	✓[c]	–	–	✓	✓	1.24±0.06
7	reductase$_{SEC}$ only	✓[d]	–	–	✓	✓	0.22±0.09
8	ferredoxin only	–	✓	–	✓	✓	–0.28±0.21
9	oxygenase only	–	✓	–	✓	✓	–0.95±0.21
10	full assay	✓	✓	✓	✓	✓	17.9±0.4

[a] All experiments were performed in MES buffer (50 mM, pH 6.8) with 100 μM $(NH_4)_2Fe(SO_4)_2$;
[b] negative rates due to temperature increase; uncertainties correspond to 95%-confidence intervals; [c] reductase purification batch without size exclusion chromatography (SEC); [d] reductase purification batch with SEC; [e] 50 μM 3-chloronitrobenzene.

We tested the consequences of background O_2 removal in the various control assays for assessing the 2NTDO-catalysed activation of O_2 by analysing the remaining fraction of O_2 for changes in $^{18}O/^{16}O$ ratios. Figure S2 illustrates that with exception of the 'no substrate' assay (entry 3 in Table S2), no change in $\delta^{18}O$ of O_2 were observed. Oxygen isotope fractionation in the 'no substrate' assay occurred to an extent that is comparable to experiments, where enzymatic O_2 activation is triggered by the presence of a substrate. Because this background reaction occurred at a > 6-fold slower rate, we exclude this source of O isotope fractionation as contribution to our experiments. The observation that the O isotope fractionation in the 'no substrate' assays were of similar magnitude as expected in the presence of the substrate suggest that some kind of oxydizable contamination might have been present in this assay. Further confirmation for neglecting such background O_2 consumptions and O isotope fractionation is shown in the following subsection.

S3.1.2 Quantification of O_2 uncoupling and background consumption

The extent of O_2 uncoupling, $f_{O_2\text{-uc}}$, was calculated through linear regressions of eq. S1 which corresponds to eq. 2 in the main manuscript.

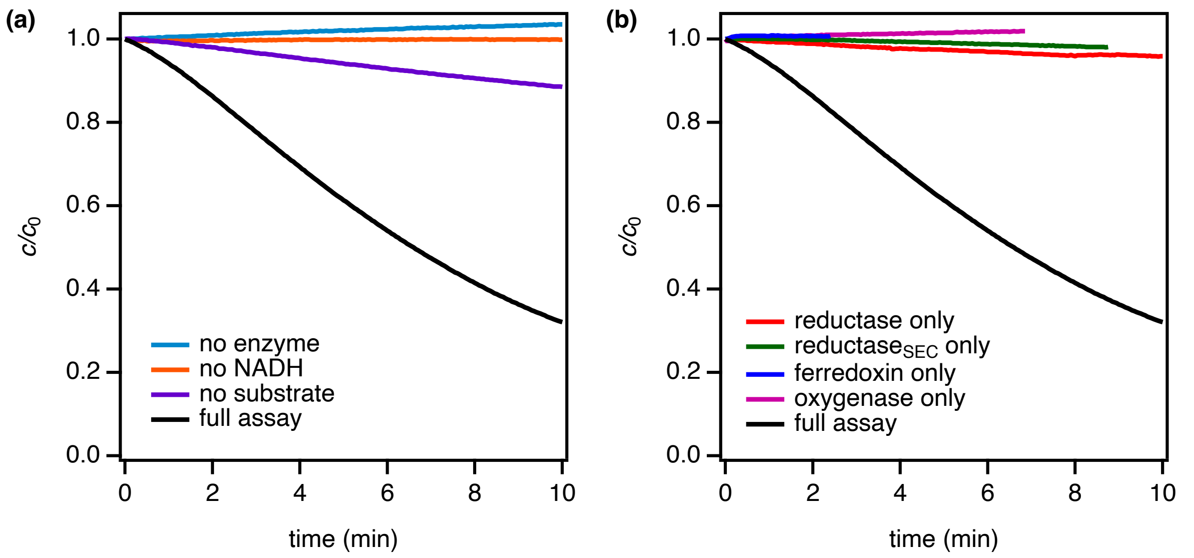

Figure S1 Trends of initial O_2 consumption determined in control experiments for 2NTDO systems. The composition of the control experiments corresponds to the entries of Table S2. Experiments were initiated by addition NADH or the start of the O_2 measurement in case of the sample without NADH.

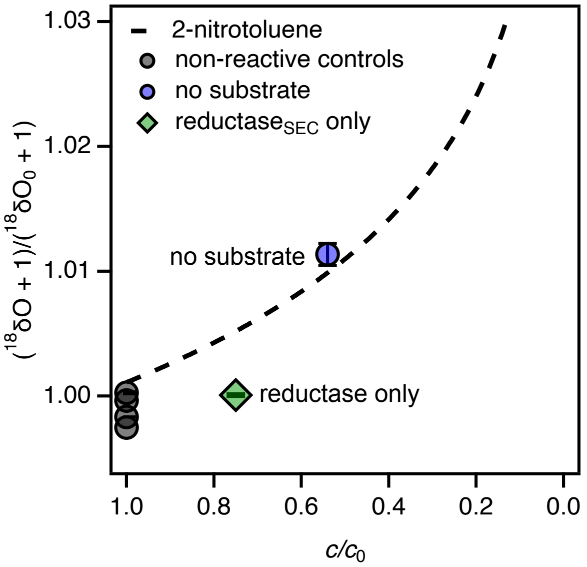

Figure S2 Oxygen isotope fractionation of O_2 in control experiments from Table S2. The dashed line illustrates a typical $^{18}O/^{16}O$ fractionation of 2-nitrotoluene. c/c_0 is the fraction of remaining of O_2.

$$
\begin{aligned}
[\mathrm{NO_2}^-] + [\mathrm{NBA}] &= (1 - f_{\mathrm{O_2\text{-}uc}}) \cdot ([\mathrm{O_2}]_0 - [\mathrm{O_2}]) + b \\
&= (1 - f_{\mathrm{O_2\text{-}uc}}) \cdot \Delta\mathrm{O_2} + b
\end{aligned} \tag{S1}
$$

where $[NO_2^-]$ is the concentration of NO_2^- formed, [NBA] is the concentration of nitrobenzylalcohol formed by monooxygenation of nitrotoluene isomers, $[O_2]_0$ and $[O_2]$ are the initial and final O_2 concentrations, and ΔO_2 is the total O_2 consumed.

Figure S3a shows linear regressions of eq. S1 for the 2NTDO-catalyzed dioxygenation of 2-nitrotoluene ($f_{O_2\text{-uc}}$ of 0.02 ± 0.03) and 4-chloronitrobenzene ($f_{O_2\text{-uc}}$ of 0.92 ± 0.01). A slope close to unity, as for 2-nitrotoluene, implies a stoichiometric transfer of activated O_2 species to the substrate and formation of (substituted) catechols. By contrast, only about 8% of the activated O_2 is used for dioxygenation in reactions with 4-chloronitrobenzene and the remainder is released as reactive oxygen species.

Figure S3b illustrates that, in some assays, the fit did not go through the origin (0|0) but had positive or negative intercepts, b, of -36 ± 2.8 to 8.22 ± 5.38 μM (Table S3). Positive intercepts are small and close to 0 within the margin of error and were not evaluated further. Negative intercepts, however, indicate a gap where a constant amount of dissolved O_2 was removed in all of the assays in one set of experiments, regardless of the NADH concentration. We interpret this gap as a O_2 background consumption, $[O_2]^{bg}$, quantified with eq. S2.

$$[O_2]^{bg} = -\frac{b}{(1 - f_{O_2\text{-uc}})} \tag{S2}$$

The data for the quantification of O_2 uncoupling including the calculated O_2 background consumption, $[O_2]^{bg}$, for all our experiments with 2NTDO and NBDO with various substrates are compiled in Table S3. Assays with selected substrates of 2NTDO gave rise considerable background consumption of O_2 with $[O_2]^{bg}$ concentrations of up to 56.5 μM (2-fluorobenzene). The origin of these O_2 losses, especially their substance dependence

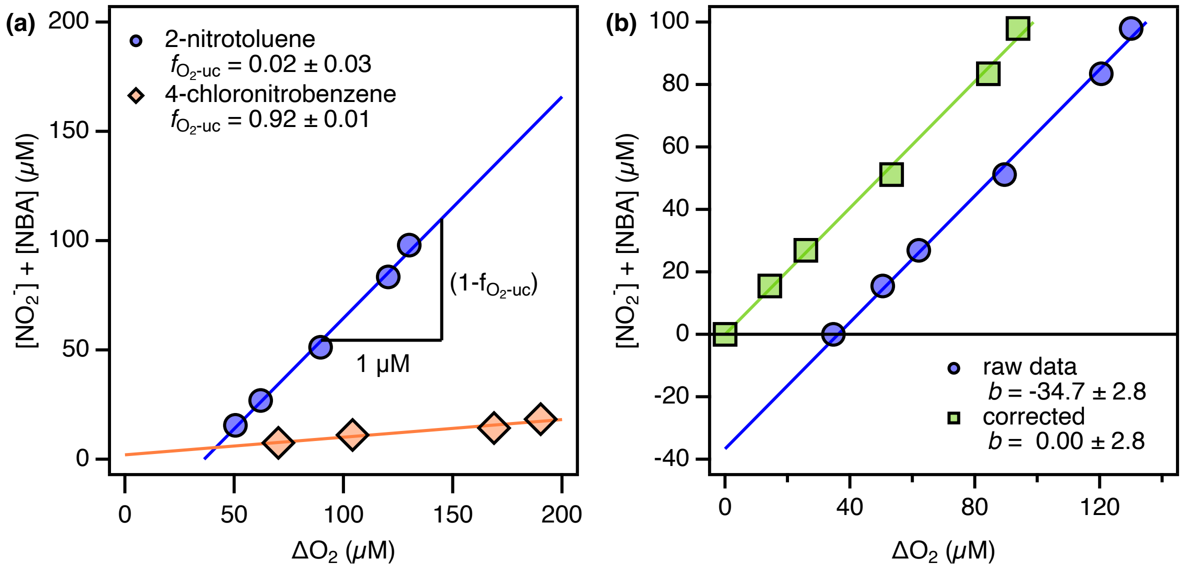

Figure S3 Quantification of O_2 uncoupling from the formation of oxygenation products vs. the consumption of O_2 in assays of 2NTDO. Lines are linear fits to eq. S1. (a) Examples two 2NTDO substrates, 2-nitrotoluene and 4-chloronitrobenzene. (b) Corrections for O_2 background consumption, $[O_2]^{bg}$, from adjusting parameter b. $[O_2]^{bg}$ is obtained from eq. S2.

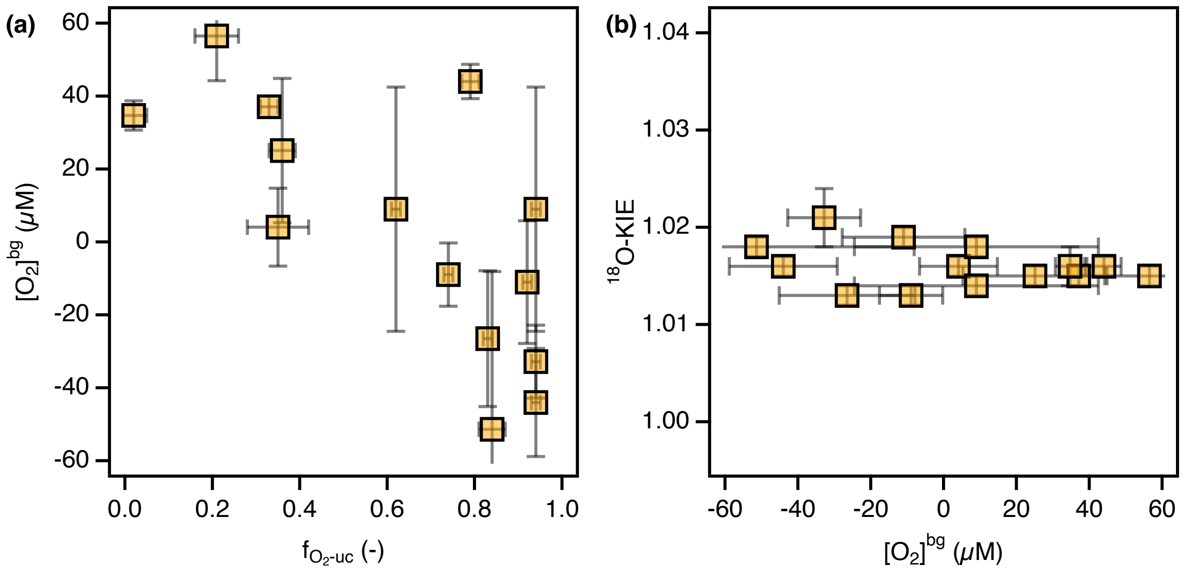

Figure S4 (a) Comparison of $[O_2]^{bg}$ with the extent of O_2 uncoupling, $f_{O_2\text{-uc}}$, in assays of 2NTDO or NBDO. (b) Comparison of ^{18}O-kinetic isotope effects of O_2 activation with the amount of O_2 background consumption, $[O_2]^{bg}$, in assays of 2NTDO or NBDO.

are unclear and no link to purification batches was observed. Qualitatively, we observed a negative trend of $[O_2]^{bg}$ with increasing $f_{O_2\text{-uc}}$ (Figure S4a). We interpret this as an artifact of the evaluation method for $f_{O_2\text{-uc}}$ (eq. S1) because measurement uncertainties are larger in the measurement of small quantities of NO_2^- and nitrobenzylalcohol and even a small systematic overestimation leads to a positive intercept, b, resulting in high negative values of $[O_2]^{bg}$. This is also supported by the fact that all negative values of $[O_2]^{bg}$ exhibit high 95% confidence intervals that make them indistinguishable from 0.

In the data evaluation, we took the phenomenon of O_2 background consumption, $[O_2]^{bg}$, into account as follows. The good quality of the linear fit (Figure S3a) is an indicator of the accuracy of the data. Inclusion of the NADH = 0 sample, where no O_2 is consumed and no NO_2^- is formed, in the raw data as (0|0), however, would distort the linear fit. Instead, we corrected the O_2 consumption, ΔO_2, by the value of $[O_2]^{bg}$ (Figure S3b). As the background consumption of O_2 is not the result of dioxygenation reactions, we excluded positive values from the calculation of $|v_{O_2}|$ with eq. 2 in the main manuscript by correcting O_2 concentrations with eq. S3.

$$[O_2]^{corr} = [O_2]^{raw} + [O_2]^{bg} \tag{S3}$$

$[O_2]^{raw}$ and $[O_2]^{corr}$ are the raw and corrected concentrations of O_2, respectively The corrected values of $|v_{O_2}|$ are indicated in Table S7 and Table 1 of the main manuscript.

The comparison of $[O_2]^{bg}$ with ^{18}O kinetic isotope effects, ^{18}O-KIEs, used for assessing the rate-limiting steps of O_2 activation, in Figure S4b shows that the O_2 background consumption did not affect this value. We speculate that the unknown process leading to background consumption of O_2 in 2NTDO assays with selected substrates might exhibit a similar ^{18}O-KIE and we refrained modifying the quantification of ^{18}O-KIEs.

Table S3 Determination of efficiency of O_2 activation, that is the share of oxygenation product per O_2 activated, $1-f_{O_2\text{-uc}}$, in assays containing 2NTDO or NBDO. b is the y-intercept from eq. S1 used for calculation of the O_2 background consumption $[O_2]^{bg}$ with eq. S2.[a]

Substrate	n^a	$1-f_{O_2\text{-uc}}{}^b$	b (μM)	$[O_2]^{bg}$ (μM)
2NTDO				
nitrobenzene[b]	7	0.67±0.02	–24.8±1.1	37.1±2.9
2-nitrotoluene[b]	6	0.98±0.03	–34.0±2.8	34.7±4.0
3-nitrotoluene[b]	4	0.16±0.03	8.22±5.38	–51.4±43.3
4-nitrotoluene[b]	7	0.06±0.01	1.97±0.27	–32.8±10.0
2-fluoronitrobenzene	5	0.64±0.03	–36.2±6.2	56.5±12.3
3-fluoronitrobenzene[c]	3	0.65±0.07	-28.6	44.0±4.7
4-fluoronitrobenzene	5	0.17±0.01	1.86±2.74	–11.0±16.8
2-chloronitrobenzene	4	0.79±0.05	–19.8±14.4	25.1±19.8
3-chloronitrobenzene	5	0.21±0.02	–0.86±2.16	4.08±10.7
4-chloronitrobenzene	5	0.08±0.01	2.12±1.22	–26.5±18.6
2-nitrophenol	4	0.06±0.01	–0.54±1.92	9.00±33.5
3-nitrophenol	5	0.00	n.a.[d]	n.a.
4-nitrophenol	7	0.06±0.01	2.64±0.45	–44.0±14.8
NBDO				
2-nitrotoluene	5	0.38±0.01	2.10±2.98	9.00±33.5
4-nitrotoluene	5	0.26±0.01	2.33±2.17	–8.96±8.69

[a] Values show results of linear regression ± 95% confidence interval weighted with measurement uncertainties; [b] number of samples including one theoretical point at (0|0); [c] unexpectedly small $f_{O_2\text{-uc}}$ led to complete consumption of the substrate in two samples;
[d] no products detected, n.a. = not applicable.

S3.2 Chemical analyses

S3.2.1 HPLC-based quantification of organic compounds

Nitroaromatic compounds, substituted catechols, substituted benzylalcohols, and 4-methoxyanilin were analyzed by reversed-phase liquid chromatography coupled to UV-vis detection at wavelengths of maximal absorption using a Dionex UltiMate 3000 System (Thermo Scientific). Samples of 10 μL were injected from an autosampler cooled to 10°C and separated either on an Xbridge BEH C18 column (50 × 3 mm, 2.5 μm, Waters) or Accucore aQ C18 column (100 × 2.1 mm, 2.6 μm, Thermo Scientific). Separation of nitroaromatic substrates, substituted catechols, and benzylalcohols was achieved with isocratic mixtures or gradients of phosphate buffer (10 mM KH_2PO_4, pH 2.5) and methanol ranging from 30% to 90% of methanol content at a flow rate of 0.5 mL min^{-1} (XBridge

BEH C18) or 0.4 mL min^{-1} (Accucore aQ c18). 4-methoxyanilin was analyzed on the XBridge BEH C18 column at an isocratic mixture of 80% phosphate buffer (10 mM KH_2PO_4, pH 7) and methanol.

S3.2.2 Quantification of dissolved O_2

Concentrations of aqueous, dissolved O_2 were measured continuously with needle-type fiber-optic oxygen microsensors connected to a 4-channel transmitter (PreSens Precision Sensing GmbH) as reported previously.[7] Up to 4 oxygen sensors were operated simultaneously after daily calibration with air-saturated and oxygen-free water. O_2 concentrations were corrected for variations in temperature. The analytical uncertainties of O_2 concentrations were smaller than ± 0.5 μM.

S3.2.3 Quantification of NO_2^- in enzyme assays

Nitrite was quantified using a photometric method[8] at 540 nm with the reagents sulfanilamide (10 g L^{-1} in 1.5 M HCl) and *N*-(1-naphthyl)ethylene diamine (1 g L^{-1} in 1.5 M HCl).

S3.3 Enzyme Kinetics

The kinetics of initial O_2 consumption and NO_2^- formation in the presence of different substrates i were evaluated in separate assays (see main manuscript). For the sake of comparability, all kinetic data were determined with the same batch of purifications. Initial rates of nitrite formation, ν^i_{0,NO_2^-}, were obtained from repeated sampling during the first 60 sec after substrate addition. Initial rates of O_2 consumption, ν^i_{0,O_2}, were determined from continuous measurements of dissolved O_2 concentration, c_{O_2}, during the first minute after NADH addition (Figure S5).

Maximum rates (ν^i_{max}) and Michaelis constants (K^i_m) of nitrite formation in the presence of different substrates i were determined with a non-linear least square regression according to eq. S4,

$$\nu^i_{0,NO_2^-} = \frac{\nu^i_{max} \cdot c^i_0}{K^i_m + c^i_0} = \frac{k^i_{cat} \cdot E_0 \cdot c^i_0}{K^i_m + c^i_0} \tag{S4}$$

where ν^i_{0,NO_2^-} is the initial rate of NO_2^- formation from substrate i, c^i_0 is the nominal initial substrate concentration, k^i_{cat} is the observable first-order rate constant, and E_0 is the nominal concentration of active sites in NBDO, corresponding to 3 mol per mol of oxygenase. By contrast, ν^i_{max} and K^i_m for O_2 consumption were obtained from the continuous measurement of O_2 concentration (c_{O_2}) over time in a single assay. The rate of O_2 consumption at each time-point ($\nu^i_{O_2}$) was calculated as the derivative of measured c_{O_2} vs. time (i.e., $\Delta[O_2]/\Delta t$). We used non-linear least square regression according to eq. S5 with the derived $\nu^i_{O_2}$ and measured $c^i_{O_2}$ values was used to estimate ν^i_{max} and K^i_m.

$$\nu^i_{O_2} = \frac{\nu^i_{max} \cdot c^i_{O_2}}{K^i_m + c^i_{O_2}} = \frac{k^i_{cat} \cdot E_0 \cdot c_{O_2}}{K^i_m + c^i_{O_2}} \tag{S5}$$

S3.4 $^{13}C/^{12}C$ ratio analysis in substrates with limited turnover

In this study, the quantification of species concentration as well as stable isotope ratios of oxygen and carbon in O_2 and organic substrates were all performed from aqueous and gaseous samples from the identical reactors. To that end, the very inefficient oxygenation of some substrates by 2NTDO and the concomitant extensive O_2 consumption compromised the quantification of their C isotope fractionation and ^{13}C-KIE values.

As shown in Table S4, the turnover of 4-nitrotoluene, as well as the three nitrophenol isomers in assays of 2NTDO, $1 - c/c_0$, was particularly low (i.e. < 0.4) or even neglible. The C isotope fractionation of these substrates was relatively minor and mostly below the total uncertainty of 0.5‰ for $^{13}C/^{12}C$ ratio measurements.[9] To that end, ^{13}C-KIE values of nitrophenol isomers were set to unity. For 4-nitrotoluene, we used the ^{13}C-KIE of 1.003 ± 0.001 determined in whole cell experiments with E. coli clones expressing 2NTDO.[10]

Table S4 Maximum substrate consumption, $1-c/c_0$, of various substrates in assays of 2NTDO and observed $^{13}C/^{12}C$ fractionation as changes in $\delta^{13}C$. $[S]_0$ is the nominal initial substrate concentration used.

Substrate	$[S]_0$ (μM)	$1 - c/c_0$	$f_{O_2\text{-uc}}$ [a]	$\Delta\delta^{13}C$ [b] (‰)
Nitrobenzene	140	0.90	0.33±0.02	3.3±0.2
2-nitrotoluene	120	0.71	0.02±0.03	0.8±0.8
3-nitrotoluene	50	0.59	0.84±0.03	0.7±0.2
4-nitrotoluene[c]	50	0.22	0.94±0.01	0.3±0.8
2-fluoronitrobenzene	120	0.80	0.36±0.03	0.0±0.4
3-fluoronitrobenzene	50	0.95	0.35±0.07	3.6±0.3
4-fluoronitrobenzene	50	0.91	0.83±0.01	0.5±0.2
2-chloronitrobenzene	150	0.80	0.21±0.05	0.1±0.6
3-chloronitrobenzene	50	0.92	0.79±0.02	4.7±0.3
4-chloronitrobenzene	50	0.37	0.92±0.01	2.1±0.4
2-nitrophenol	150	0.14	0.94±0.01	0.8±0.4
3-nitrophenol	150	1.00	1.00	0.4±0.3
4-nitrophenol	150	0.16	0.94±0.01	–0.3±0.4

[a] Values show results of linear regression ± 95% confidence interval weighted with measurement uncertainties;
[b] value ± standard deviation of triplicate measurements;
[c] $\delta^{13}C$ values originate from a separate experiment.

S4 Additional Results

S4.1 Survey of initial rates of O_2 consumption rates by various substrate of 2NTDO

We surveyed the reactivity of 2NTDO towards various aromatic substrates by examining the O_2 consumption in small scale experiments (see experiment description in the main manuscript). Time courses of normalized O_2 concentrations and inital rates of O_2 consumption, ν_0, are shown in Figure S5 and Table S5.

We observed three distinct types of substrate-dependent behaviour. First, various well-known nitroaromatic substrates consume O_2 with concomitant generation of NO_2^- from the dioxygenation reaction. Their O_2 consumption is shown in Figure S5a. These are summarized as type-1-substrates in Table S5 and include new substrates such as dichloronitrobenzene and nitronaphthalene. Second, we identified nitroaromatic substrates that cause O_2 disappearance without generation of NO_2^-. These substrates, summarized as type-2-substrates in Table S5 include the explosives 2,4,6-trinitrotoluene and 2,4-dinitroanisole which, like nitrophenols, act as O_2 uncoupling compounds. Finally, we find that the O_2 consumption of aromatic compounds lacking a NO_2-group do not consume any O_2 beyond background losses. These compounds include benzene, toluene, naphthalene, and benzoate and no efforts were made to measure any of the expected dioxygenation products.

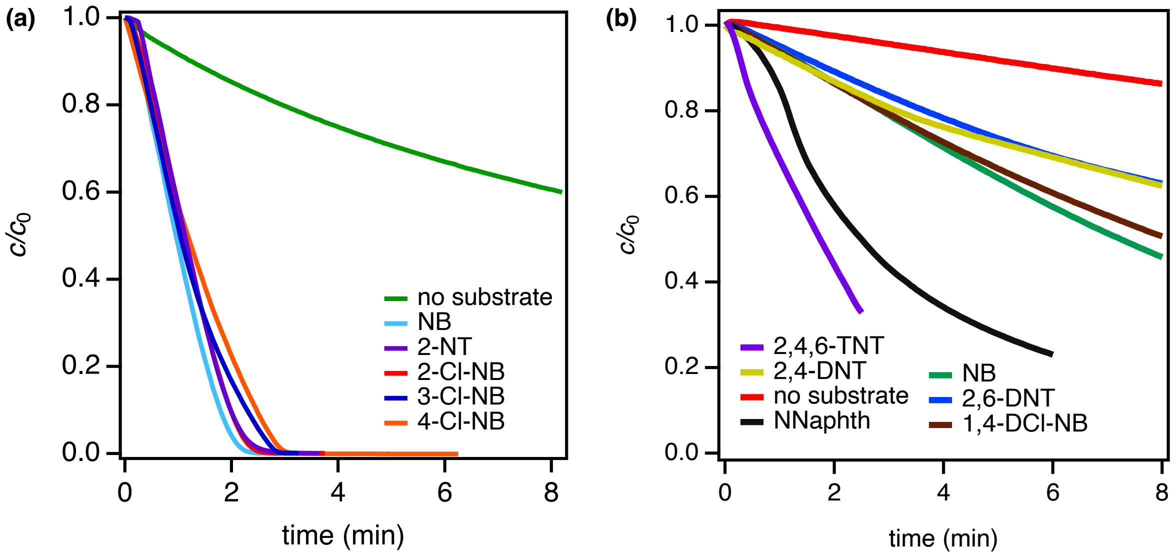

Figure S5 Normalized decrease of dissolved O_2 concentrations in 2NTDO assays without headspace with different substrates. (a) O_2 consumption in 2NTDO assays for the determination of O_2 consumption kinetics as described in the experiment section of the main manuscript. (b) O_2 consumption in 2NTDO assays with 0.15 μM reductase, 1.8 μM ferredoxin, 0.15 μM oxygenase, 100 μM $(NH_4)_2Fe(SO_4)_2$, 150 μM substrate, and 250 μM NADH (Initial rates of O_2 consumption are shown in Table S5).

Table S5 Initial rates of O_2 consumption by 2NTDO in the presence of different substrates ordered by magnitude. O_2 consumption was determined in 2NTDO assays with 0.15 μM reductase, 1.8 μM ferredoxin, 0.15 μM oxygenase, 100 μM $(NH_4)_2Fe(SO_4)_2$, 150 μM substrate, and 250 μM NADH.[a]

Substrate	Abbrev.	ν_0 (μM min^{-1})	NO_2^- (μM)
Type 1 substrates			
nitrobenzene	NB	18.8±0.2	102
2-fluoronitrobenzene	2-F-NB	19.4±0.4	89
3-fluoronitrobenzene	3-F-NB	26.4±0.2	44
4-fluoronitrobenzene	4-F-NB	12.0±0.3	35
2-chloronitrobenzene	2-Cl-NB	27.3±2.4	111
3-chloronitrobenzene	3-Cl-NB	29.7±0.5	12
4-chloronitrobenzene	4-Cl-NB	16.3±0.3	6.7
4-nitrophenol	4-OH-NB	13.4±0.4	26
2,6-dinitrotoluene	2,6-DNT	16.4±0.2	12
1,4-dichloro-2-nitrobenzene	1,4-DCl-NB	20.4±0.2	34
1-nitronaphthalene	NNaphth	66.0±5.7	9.0
Type 2 substrates			
2-nitrophenol	2-OH-NB	34.0±0.6	0
3-nitrophenol	3-OH-NB	18.0±0.6	0
2,4,6-trinitrotoluene	2,4,6-TNT	82.4±4.1	0
2,4-dinitrotoluene	2,4-DNT	17.2±0.4	0
2,4-dinitroanisole	2,4-DNAN	9.69±0.13	0

[a] Uncertainties correspond to 95%-confidence intervals.

S4.2 H_2O_2 quantification

We quantified H_2O_2 concentrations in enzyme assays based on horse radish peroxidase catalyzed turnover of either Ampliflu™ or *p*-methoxyanilin. Results are presented in Table S6.

Table S6 H_2O_2 concentration measured in enzyme assays. Data are single measurements from separate experimental assays.

Substrate	reducing agent	ΔO_2 (μM)	NO_2^- (μM)	$f_{O_2\text{-uc}}$ [a]	H_2O_2 (μM)	$H_2O_2/\Delta O_2$	$H_2O_2/(\Delta O_2–NO_2^-)$
2-nitrotoluene dioxygenase							
nitrobenzene	Ampliflu	194	127	0.35	0.0	0.00	0.00
2-nitrotoluene	Ampliflu	183	126	0.31	16	0.09	0.29
2-chloronitrobenzene	Ampliflu	188	117	0.38	2.1	0.01	0.03
3-chloronitrobenzene	Ampliflu	186	23	0.88	36	0.19	0.22
4-chloronitrobenzene	Ampliflu	182	9.3	0.95	79	0.43	0.46
nitrobenzene dioxygenase							
2-nitrotoluene	*p*-methoxyanilin	153	35	0.77	0.0	0.0	0.00
4-nitrotoluene	*p*-methoxyanilin	138	36	0.74	35	0.25	0.34

[a] Determined for single assay as $f_{O_2\text{-uc}} = 1 - NO_2^-/\Delta O_2$.

S4.3 Stoichiometric coefficients of substrate consumption and product formation

Table S7 Stoichiometric coefficients, $|\upsilon_j|$ of substrate (S) and O_2 consumption as well as formation of NO_2^-, nitrobenzylalcohols (NBA), and total hydroxylated aromatic products (P) per nominal concentration of NADH for transformation of nitroaromatic substrates by 2NTDO and NBDO. 3-P and 4-P refer to *meta*- and *para*- substituted catechols, respectively. [a]

**Substrate**	$\lvert\upsilon_S\rvert$	$\lvert\upsilon_{O_2}\rvert$	$\lvert\upsilon_{NO_2^-}\rvert$	$\lvert\upsilon_P\rvert$[b]	$\lvert\upsilon_{3\text{-}P}\rvert$[c]	$\lvert\upsilon_{4\text{-}P}\rvert$[d]	$\lvert\upsilon_{NBA}\rvert$
2NTDO							
nitrobenzene[b]	0.47 ± 0.01	0.65 ± 0.01[e]	0.50 ± 0.02	0.42 ± 0.02	n.a.[f]	n.a.	n.a.
2-nitrotoluene[b]	0.55 ± 0.02	0.63 ± 0.01[e]	0.62 ± 0.02	0.63 ± 0.02	0.63 ± 0.02	n.a.	0.03 ± 0.01[g]
3-nitrotoluene[b]	0.27 ± 0.03	0.99 ± 0.01	0.16 ± 0.02	0.10 ± 0.01	0.09 ± 0.01	0.03 ± 0.01	0.02 ± 0.01[h]
4-nitrotoluene[b]	0.10 ± 0.03	0.85 ± 0.01	0.05 ± 0.01	0.01 ± 0.01	n.a.	0.01 ± 0.01	n.d.[i,j]
2-fluoronitrobenzene	0.47 ± 0.02	0.68 ± 0.01[e]	0.40 ± 0.02	0.31 ± 0.01	0.31 ± 0.01	n.a.	n.a.
3-fluoronitrobenzene	0.41 ± 0.02	0.62 ± 0.01[e]	0.44 ± 0.03	0.25 ± 0.03	0.04 ± 0.01	0.22 ± 0.03	n.a.
4-fluoronitrobenzene	0.15 ± 0.01	0.79 ± 0.01	0.13 ± 0.01	0.02 ± 0.01	n.a.	0.02 ± 0.01	n.a.
2-chloronitrobenzene	0.64 ± 0.05	0.79 ± 0.01[e]	0.66 ± 0.05	0.53 ± 0.04	0.53 ± 0.04	n.a.	n.a.
3-chloronitrobenzene	0.09 ± 0.01	0.51 ± 0.01[e]	0.10 ± 0.01	0.03 ± 0.01	0.01 ± 0.01	0.03 ± 0.01	n.a.
4-chloronitrobenzene	0.04 ± 0.01	0.59 ± 0.01	0.04 ± 0.01	0.01 ± 0.01	n.a.	0.01 ± 0.01	n.a.
2-nitrophenol	0.23 ± 0.09	1.09 ± 0.01[e]	0.07 ± 0.01	n.m.[k]	n.m.	n.a.	n.a.
3-nitrophenol	0.17 ± 0.06[l]	1.07 ± 0.01	n.d.	n.m.	n.m.	n.m.	n.a.
4-nitrophenol	0.07 ± 0.02	0.66 ± 0.01	0.04 ± 0.01	n.m.	n.m.	n.m.	n.a.
NBDO							
2-nitrotoluene	0.39 ± 0.06	0.89 ± 0.01	0.18 ± 0.02[m]	0.10 ± 0.01[m]	0.10 ± 0.01[m]	n.a.	0.15 ± 0.01[g]
4-nitrotoluene	0.28 ± 0.06	0.80 ± 0.01	0.18 ± 0.02[m]	0.10 ± 0.01[m]	n.a.	0.10 ± 0.01[m]	0.02 ± 0.01[i]

[a] Quantification on the basis of eq. 2 in the main manuscript, uncertainties correspond to 95%-confidence intervals weighted with measurement uncertainties; [b] catechol (nitrobenzene) or sum of *meta*- and *para*-substituted catechols; [c] *meta*-substituted catechol; [d] *para*-substituted catechol; [e] without O_2 background consumption according to eq. S2; [f] n.a. = not applicable; [g] 2-nitrobenzylalcohol; [h] 3-nitrobenzylalcohol; [i] 4-nitrobenzylalcohol; [j] n.d. = not detected; [k] n.m. = not measured; [l] substrate concentration declined without formation of NO_2^-; [m] rounded values are indeed identical.

S4.4 Oxygen isotope fractionation of dissolved O_2

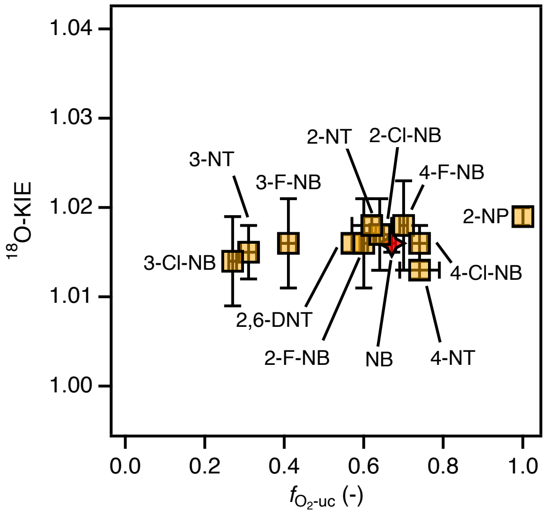

Figure S6 ^{18}O-Kinetic isotope effects of O_2 activation by NBDO in the presence of various nitroaromatic substrates vs. extent of O_2 uncoupling, $f_{O_2\text{-uc}}$. Data set reproduced in part form Pati et al.[1] and extended for 2- and 4-nitrotoluene.

S4.5 Carbon isotope fractionation nitroaromatic substrate hydroxylation

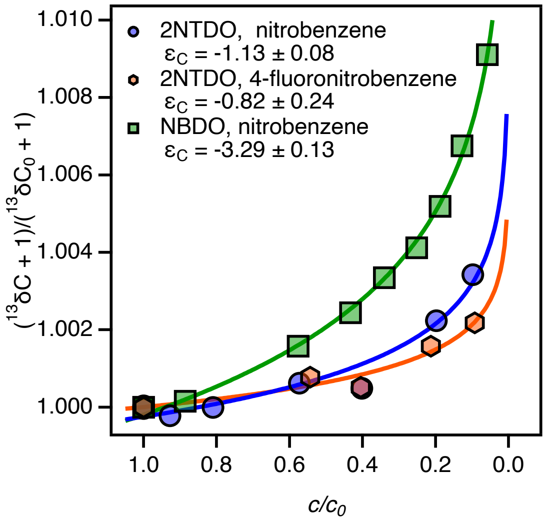

Figure S7 Normalized carbon isotope fractionation associated with the hydroxylation of nitrobenzene and 4-fluoronitrobenzene by NBDO and 2NTDO, respectively. Data are presented according to eq. 3 of the main manuscript. The data set for nitrobenzene and NBDO is reproduced here from Pati et al.[11]

S4.6 Enzyme Kinetics

Table S8 Kinetic parameters for the dioxygenation of nitroaromatic substrate (S) dioxygenation and O_2 activation by 2NTDO and NBDO. [a]

**substrate**	**species**	$\nu_{\mathbf{max}}$ (μM s^{-1})	k_{cat} (s^{-1})	K_m (μM)	k_{cat}/K_m (10^3 M^{-1} s^{-1})	ν_0 (μM min^{-1})
2NTDO						
nitrobenzene	(S)	0.83 ± 0.53	1.84 ± 1.17	15.0 ± 23.8	123 ± 274	n.a.[b]
	(O_2)	2.39 ± 0.11	5.31 ± 0.23	16.6 ± 3.9	320 ± 89	143 ± 1
2-nitrotoluene	(S)	1.06 ± 0.51	2.36 ± 1.13	36.0 ± 38.4	66 ± 102	n.a.
	(O_2)	2.79 ± 0.03	6.19 ± 0.06	31.1 ± 1.2	199 ± 9	140 ± 1
2-chloronitrobenzene	(O_2)	2.29 ± 0.02	5.09 ± 0.05	18.9 ± 0.9	269 ± 16	121 ± 2
3-chloronitrobenzene	(S)	0.55 ± 0.22	1.22 ± 0.48	75.2 ± 65.8	16 ± 21	n.a.
	(O_2)	2.39 ± 0.24	5.31 ± 0.52	61.7 ± 17.0	86 ± 32	133 ± 1
4-chloronitrobenzene[c]	(O_2)	1.37 ± 0.05	3.05 ± 0.11	19.0 ± 3.0	164 ± 32	75.3 ± 0.7
NBDO						
2-nitrotoluene	(O_2)	0.75 ± 0.01	1.67 ± 0.03	45.7 ± 2.6	37 ± 3	35.1 ± 0.3
4-nitrotoluene	(O_2)	0.97 ± 0.04	2.15 ± 0.09	47.1 ± 6.6	46 ± 8	49.5 ± 0.6

[a] Substrate dioxygenation kinetics quantified on the basis of NO_2^- formation as in eq. S4, kinetics of O_2 consumption derived from eq. S5, all uncertainties correspond to 95%-confidence intervals, additional data for 10 additional substrates of NBDO are presented in Table S3 of Pati et al.[1]; [b] n.a. = not applicable;
[c] insufficient substrate turnover for substrate kinetic experiment.

References

[1] Pati, S. G.; Bopp, C. E.; Kohler, Hans-Peter E,; Hofstetter, T. B. Substrate-specific coupling of O_2 activation to hydroxylation of aromatic compounds by Rieske nonheme iron dioxygenases. *ACS Catal.* **2022**, *12*, 6444–6456, https://doi.org/10.1021/acscatal.2c00383.

[2] Parales, R. E.; Huang, R.; Yu, C. L.; Parales, J. V.; Lee, F. K. N.; Lessner, D. J.; Ivkovic-Jensen, M. M.; Liu, W.; Friemann, R.; Ramaswamy, S.; Gibson, D. T. Purification, characterization, and crystallization of the components of the nitrobenzene and 2-nitrotoluene dioxygease enzyme systems. *Appl. Environ. Microbiol.* **2005**, *71*, 3806–3814, https://doi.org/10.1128/AEM.71.7.3806-3814.2005.

[3] Mahan, K.; Penrod, J.; Ju, K.; Al Kass, N.; Tan, W.; Truong, R.; Parales, J.; Parales, R. Selection for growth on 3-nitrotoluene by 2-nitrotoluene-utilizing Acidovorax sp. strain JS42 identifies nitroarene dioxygenases with altered specificities. *Appl Environ Microbiol* **2015**, *81*, 309–319, http://dx.doi.org/10.1128/aem.02772-14.

[4] Pati, S. G.; Kohler, H.-P. E.; Bolotin, J.; Parales, R. E.; Hofstetter, T. B. Isotope Effects of Enzymatic Dioxygenation of Nitrobenzene and 2-Nitrotoluene by Nitrobenzene Dioxygenase. *Environ. Sci. Technol.* **2014**, *48*, 10750–10759, http://dx.doi.org/10.1021/es5028844.

[5] Lessner, D. J.; Johnson, G. R.; Parales, R. E.; Spain, J. C.; Gibson, D. T. Molecular characterization and substrate specificity of nitrobenzene dioxygenase from *Comamonas* sp strain JS765. *Appl. Environ. Microbiol.* **2002**, *68*, 634–641, https://doi.org/10.1128/aem.68.2.634-641.2002.

[6] Burgess, R. R. In *Guide to Protein Purification*, 2nd ed.; Burgess, R. R., Deutscher, M. P., Eds.; Methods in Enzymology; Academic Press, 2009; Vol. 463; Chapter Chapter 4, pp 29–34, https://doi.org/10.1016/S0076-6879(09)63004-4.

[7] Spahr, S.; Cirpka, O. A.; von Gunten, U.; Hofstetter, T. B. Formation of *N*-nitrosodimethylamine during chloramination of secondary and tertiary amines: Role of molecular oxygen and radical intermediates. *Environ. Sci. Technol.* **2017**, *51*, 280–290, https://doi.org/10.1021/acs.est.6b04780.

[8] An, D.; Gibson, D. T.; Spain, J. C. Oxidative release of nitrite from 2-nitrotoluene by a three-component enzyme system from *Pseudomonas* sp. strain JS42. *J. Bacteriol.* **1994**, *176*, 7462–7467, https://doi.org/10.1128/jb.176.24.7462-7467.1994.

[9] Lollar, B. S.; Hirschorn, S.; Chartrand, M.; Lacrampe-Couloume, G. An approach for assessing total instrumental uncertainty in compound-specific carbon isotope analysis: Implications for environmental remediation studies. *Anal. Chem.* **2007**, *79*, 3469–3475, https://doi.org/10.1021/ac062299v.

[10] Pati, S. G.; Kohler, H.-P. E.; Pabis, A.; Paneth, P.; Parales, R. E.; Hofstetter, T. B. Substrate and enzyme specificity of the kinetic isotope effects associated with the dioxygenation of nitroaromatic contaminants. *Environ. Sci. Technol.* **2016**, *50*, 6708–6716, http://dx.doi.org/10.1021/acs.est.5b05084.

[11] Pati, S. G.; Kohler, H.-P. E.; Hofstetter, T. B. In *Measurement and Analysis of Kinetic Isotope Effects*; Harris, M. E., Anderson, V. E., Eds.; Academic Press, 2017; pp 292–329, https://doi.org/10.1016/bs.mie.2017.06.044.
